# Bruton’s tyrosine kinase drives neuroinflammation and anxiogenic behavior in mouse models of stress

**DOI:** 10.1186/s12974-021-02322-9

**Published:** 2021-12-11

**Authors:** Simantini Ghosh, Zaidan Mohammed, Itender Singh

**Affiliations:** 1 Department of Neurology, Washington University School of Medicine in St. Louis, St. Louis, MO USA; 2 Department of Psychology, Ashoka University, Rai, India; 3 Department of Neurosurgery, Washington University School of Medicine in St. Louis, St. Louis, MO USA; 4 Ambedkar Center for Biomedical Research, Delhi University, New Delhi, India

**Keywords:** Bruton’s tyrosine kinase, NLRP3 inflammasome, Neuroinflammation, Posttraumatic stress disorder, Ibrutinib, LFM-A13, Stress, Predator odor stress, Physical restraint stress, Systemic inflammation

## Abstract

**Background:**

Current therapies targeting several neurotransmitter systems are only able to partially mitigate the symptoms of stress- and trauma-related disorder. Stress and trauma-related disorders lead to a prominent inflammatory response in humans, and in pre-clinical models. However, mechanisms underlying the induction of neuroinflammatory response in PTSD and anxiety disorders are not clearly understood. The present study investigated the mechanism underlying the activation of proinflammatory NLRP3 inflammasome and IL1β in mouse models of stress.

**Methods:**

We used two mouse models of stress, i.e., mice subjected to physical restraint stress with brief underwater submersion, and predator odor stress. Mice were injected with MCC950, a small molecule specific inhibitor of NLRP3 activation. To pharmacologically inhibit BTK, a specific inhibitor ibrutinib was used. To validate the observation from ibrutinib studies, a separate group of mice was injected with another BTK-specific inhibitor LFM-A13. Seven days after the induction of stress, mice were examined for anxious behavior using open field test (OFT), light–dark test (LDT), and elevated plus maze test (EPM). Following the behavior tests, hippocampus and amygdale were extracted and analyzed for various components of NLRP3–caspase 1–IL1β pathway. Plasma and peripheral blood mononuclear cells were also used to assess the induction of NLRP3–Caspase 1–IL-1β pathway in stressed mice.

**Results:**

Using two different pre-clinical models of stress, we demonstrate heightened anxious behavior in female mice as compared to their male counterparts. Stressed animals exhibited upregulation of proinflammatory IL1β, IL-6, Caspase 1 activity and NLRP3 inflammasome activation in brain, which were significantly higher in female mice. Pharmacological inhibition of NLRP3 inflammasome activation led to anxiolysis as well as attenuated neuroinflammatory response. Further, we observed induction of activated Bruton’s tyrosine kinase (BTK), an upstream positive-regulator of NLRP3 inflammasome activation, in hippocampus and amygdala of stressed mice. Next, we conducted proof-of-concept pharmacological BTK inhibitor studies with ibrutinib and LFM-A13. In both sets of experiments, we found BTK inhibition led to anxiolysis and attenuated neuroinflammation, as indicated by significant reduction of NLRP3 inflammasome and proinflammatory IL-1β in hippocampus and amygdala. Analysis of plasma and peripheral blood mononuclear cells indicated peripheral induction of NLRP3–caspase 1–IL1β pathway in stressed mice.

**Conclusion:**

Our study identified BTK as a key upstream regulator of neuroinflammation, which drives anxiogenic behavior in mouse model of stress. Further, we demonstrated the sexually divergent activation of BTK, providing a clue to heightened neuroinflammation and anxiogenic response to stress in females as compared to their male counterparts. Our data from the pharmacological inhibition studies suggest BTK as a novel target for the development of potential clinical treatment of PTSD and anxiety disorders. Induction of pBTK and NLRP3 in peripheral blood mononuclear cells of stressed mice suggest the potential effect of stress on systemic inflammation.

**Supplementary Information:**

The online version contains supplementary material available at 10.1186/s12974-021-02322-9.

## Background

Posttraumatic stress disorder (PTSD) is characterized by an impaired stress response comorbid with severe anxiety among other symptoms [1]. PTSD is a common finding after exposure to occupational and personal traumatic events leading to immense health and socioeconomic ramifications [2]. According to one estimate globally 345 million adult war survivors are likely to have experienced some PTSD symptoms [3], and this statistic does not take in to account any other trauma type, including sexual violence, which is often as potent a contributor to PTSD as war exposure [1, 4]. Present therapies of PTSD are often symptomatic, and fall short of treating PTSD and preventing relapse in long term [2, 5–7]. It is well documented that PTSD is associated with a chronic proinflammatory state, characterized by an elevated secretion of IL1β, IL-6, TNF-α, and other inflammatory mediators by the monocytes in the blood and microglia in the brain [8–11]. The data on whether existing selective serotonin reuptake inhibitors (SSRI) or serotonin–norepinephrine reuptake inhibitors (SNRI)-based pharmacological treatment regimens can ameliorate this neuroinflammatory phenotype are inconclusive at best [12–14]. However, the therapeutic potential of targeting the chronically active neuroinflammatory pathways has not yet been investigated thoroughly. Females are more prone to PTSD, and symptoms of anxiogenic behavior and neuroinflammation are more severe in females as compared to males. However, mechanisms underlying the sexually divergent stress response and neuroinflammation are not understood well in PTSD and anxiety disorders.

In this context, we set out to investigate the potential mechanisms underlying the sexually divergent neuroinflammation profile in stress disorders. As a first step, we looked at the involvement of the multi-protein nucleotide-binding oligomerization domain (NOD) like receptor (NLR) family, pyrin domain containing 3 (NLRP3) inflammasome complex in male and female mice in stress models. The NLRP3 inflammasome complex is a pattern recognition receptor of the innate immune system expressed widely in the central nervous system and cells of myeloid lineage [15–19]. Inflammasomes evolved to act as host sensors to pathogen as well as endogenous stress, and work by the way of activating Caspase 1, which leads to the production of the pleiotropic cytokine IL1β and further neuroinflammatory sequelae [20–22]. IL1β in particular has been previously described as a major driver of the proinflammatory response in the brain and extensively investigated in the context of stroke as well as several neurodegenerative disorders [23–26]. The contribution of NLRP3 activation towards pathophysiology and behavioral deficits in neurocognitive disorders and psychiatric disorder has emerged as an active area of investigation [27–30]. In the present study, for the first time, we demonstrate that there is a sexually divergent induction of NLRP3 post stress. The female stressed mice, as compared to their male counterparts, not only showed heightened anxious behavior, but also significantly increased upregulation of NLRP3 in hippocampus and amygdala. Inhibition of NLRP3 with a small molecule inhibitor MCC950 significantly reduced stress-induced anxiety in mice and IL1β as well as Caspase 1 activity. Recent independent observation from other groups [31–33] also showed that pharmacological inhibition of NLRP3 using MCC950 [34] reduced anxiety behavior in rodents.

Thereafter, we focused on our primary goal, i.e., Bruton’s Tyrosine Kinase (BTK), an molecule hitherto unrecognized in literature pertinent to PTSD and other anxiety disorder. Recently, BTK was identified as an upstream regulator of NLRP3 activation in a few models of inflammation [35, 36]. However, there is no study linking BTK to any stress disorders. We hypothesized that activation of BTK induces the NLRP3 inflammasome, which initiates the maturation of master regulator cytokines such as IL1β, thus driving the vicious cycle of self-synthesis and further neuroinflammation. Therefore, we worked with the hypothesis that if BTK could be inhibited, then we would see a rescue from stress-induced anxiety with the reversal of the neuroinflammatory profile. In the present study, first we demonstrate the activation of BTK in hippocampus and amygdala of stressed mice. The female stressed mice exhibited significantly higher activation of BTK, which potentially provides a clue to higher degree of activation of downstream neuroinflammatory mediators such as NLRP3 and IL1β in stressed females. Thereafter, to inhibit BTK, we employ ibrutinib, a BTK inhibitor that is FDA approved for human therapeutic use in certain types of leukemia and lymphomas [37, 38]. Ibrutinib-treated mice show marked reduction in stress-induced anxiety, IL1β levels and Caspase 1 activity in the hippocampus and amygdala. We performed an orthogonal validation experiment with a second pharmacological inhibitor of BTK, LFM-A13, to observe similar results. Several clinical and experimental studies in the past have demonstrated the induction of systemic inflammation following stress [39–41]. In the present study, we observed the induction of IL1β in plasma, and p-BTK, NLRP3 and Caspase 1 in peripheral blood mononuclear cells (PBMC) from stressed mice. Further, inhibition of BTK with ibrutinib in stressed mice attenuated plasma IL1β levels, suggesting  a potential role of BTK in systemic inflammation following stress.

The key finding of the present study is the identification of BTK as an upstream regulator of NLRP3 and other neuroinflammatory mediators, which contributes to anxiogenic behavior in stress disorders.

## Material and methods

### Experimental animals

Male and female wild-type C57BL/6 mice were obtained from Jackson Laboratories (Bar Harbor, ME). They were housed in vivarium under standard conditions (12 h: 12 h light–dark cycle starting at 7 am; 74°F; 55 ± 10% humidity) in solid-bottomed cages on woodchips bedding and had free access to autoclaved water and chow. All studies were performed according to the National Institute of Health guidance using protocols approved by Animal Studies Committees at Washington University School of Medicine and University of Delhi. Age-matched 14-week-old male and female C57BL/6 mice were used for experiments.

### Inhibitors

To pharmacologically inhibit NLRP3 inflammasome, a specific inhibitor MCC950 (Sigma-Aldrich, St. Louis, MO) was injected (IP: 50 mg/Kg) as reported in [42], starting 72 h before the induction of stress in mice, and again given at every 24 h until the end of the experiment. Control mice were similarly injected with vehicle (0.05% DMSO in saline, i.p.).

To pharmacologically inhibit BTK, a specific inhibitor ibrutinib (PCI-32765, Abcam, Cambridge, MA) was administered (IP: 3 mg/Kg) starting 72 h before the induction of stress in mice, and again given at every 24 h until the end of the experiment. Control mice were injected in parallel with the vehicle (0.05% DMSO in saline, i.p.) at the same time points. To validate the observation from ibrutinib studies, a separate group of mice was injected (IP: 50 mg/kg) with another BTK-specific inhibitor LFM-A13 (Sigma-Aldrich) at similar time points as mentioned above. Control mice were similarly injected with vehicle (0.05% DMSO in saline, i.p.).

To determine the potential therapeutic efficacy of ibrutinib in post-stress paradigm, mice were injected daily with ibrutinib (IP: 3 mg/kg) 2 days after induction of stress in mice. Control mice were injected in parallel with the vehicle (0.05% DMSO in saline, i.p.) at the same time points. Ibrutinib and vehicle were administered daily for 5 days.

### Stress behavioral paradigm

Two weeks before behavioral testing, mice were gently handled daily. Mice were picked from the tail and permitted to explore freely on an investigator's gloved hand for 2 min. Mice were subjected to predator odor stress to induce psychological stress or restraint stress to induce physical stress. All stress induction experiments were performed at night between 8 pm and 3 am to avoid potential influence from noise and disturbances from day time activities.

To induce psychological stress, mice (prey) were placed away from the home cage in empty black plexiglass box (45 × 45 × 45 cm) containing fresh rat (predator) feces for 3 h. The plexiglass box, as well as gloved hands, were wiped clean with 70% ethanol solution and let dry between tests with each mouse to remove any olfactory cues to mice. Since predator smell can come to the testing room and influence other animals; care was taken to expose the mice to rat feces under a fume hood.

To induce physical stress, mice were restrained by immobilizing them in disposable plastic tubes (4 cm – diameter × 12 cm – length) containing perforations near the nose for easy breathing and other ends with a hole in the closing cap for the tail to come out. Restrained mice were placed in a plexiglass box (45 × 45 × 45 cm) under the fume hood for 3 h. Thereafter mice were immediately placed in a container with ice-cold water and allowed to swim for 45 s, and then gently submerged completely underwater for 30 s. Restraining plastic tubes were used only once for each mouse. The plexiglass box as well as gloved hands were carefully cleaned with 70% ethanol solution and let dry between each session.

Control mice were placed similarly in separate plexiglass boxes (45 × 45 × 45 cm) near the stress experiment arena, however, away from the fume hood. Control exposures were completed before starting the stress experiments to avoid any potential disturbance to the control of mice from the stress paradigm. As an additional control and to see the potential influence of control mice from the surrounding in the stress experiment arena, a set of control mice were kept in a vivarium away from the stress experimental arena.

### Anxiety behavior

Seven days after the induction of predator odor and physical stress, the stressed and non-stressed control mice were examined for anxious behavior using open field test (OFT), light–dark test (LDT), and elevated plus maze test (EPM). All behavior assessment experiments were performed at night between 8 pm and 3 am when rodents are most active—being nocturnal, and also to avoid potential influence from noise and disturbances from day time activities. Between tests with each mouse, all the behavior tools and hands were thoroughly wiped clean with 70% ethanol and dried well to remove any olfactory cues from the experimental mice. Behavior tests were performed in the following sequence: open field test, light–dark test, and the elevated plus maze with 1 h interval between each test. These tests represent a standard battery utilized for the examination of anxious behavior [43, 44].

### Open field test

Examination of anxious behavior of stressed and non-stressed control mice was conducted using an open-aired rectangular gray-colored opaque box (30 × 45 cm) surrounded by a 35 cm high wall. At the beginning of the test, each mouse was placed into the same left spot at one corner of the rectangular arena and allowed to freely explore the arena for 7 min. The time spent in the central arena – marked by an 8 × 12 cm rectangle was measured to assess anxiogenic behavior. Stressed animals tend to show hesitation to spend time in a central area away from the sidewalls of the box [45]. The testing arena was cleaned with 70% ethanol before and between each trial.

### Light–dark test

This test utilizes a light–dark box to examine the innate tendency of mice to avoid the brightly lit area and escape to the safety of a dark area, especially when stressed [46, 47]. The test box consisted of a rectangular plexiglass box (50 × 30 cm) with one-half enclosed with black opaque plexiglass sheet to serve as a dark area and the other half had a clear plexiglass enclosure to act as a light area. These two areas were connected with an opening of 8 × 8 cm to enable the mouse to freely move in these areas. Mice were let in the box for 10 min and time spent in each chamber was counted to assess the anxiety behavior in mice. The light–dark box was cleaned thoroughly with 70% ethanol between each trial to remove any olfactory cues for the mice.

### Elevated plus maze

After OFT and LDT, mice were subjected to elevated plus maze tests based on previously published methods [48, 49]. Elevated plus maze was made of four perpendicular plus-shaped arms of plexiglass (30 cm in length, 7 cm in width), extending from the central square (7cm^2^) at the elevation of 60 cm from the floor. One set of opposing arms had 15 cm high walls—representing a secure area, and the other set of opposing arms were without walls—representing an unsafe area. Elevated plus maze is based on the innate aversion of mice to open elevated unsafe spaces, and uses the conflict between exploration and this aversion. Mice were introduced into the central square of the elevated maze and allowed to freely explore all the arms for 5 min. Anxious behavior was determined by the percent time spent in the open arm of the elevated plus maze. An arm entry was counted when all the four limbs of the mouse enter into the open arm. Increased time spent in the open arm denotes a lower degree of anxiety in the rodents [49].

### Tissue harvesting

Following the behavior tests, mice were anesthetized by injection with a mixture of ketamine (100 mg/kg) and xylazine (10 mg/Kg) and transcardially perfused with ice-cold heparinized phosphate-buffered saline (PBS) to clear the brain vasculature of blood and peripheral immune cells [50, 51]. The brains were quickly extracted, hemi-dissected, and hippocampus and amygdale isolated under the stereomicroscope. Tissues were snap-frozen in liquid nitrogen and stored in a − 80 °C freezer for later use.

### Collection of peripheral blood mononuclear cells

About 1 ml of mouse blood was collected from cardiac puncture. Separation and extraction of mouse peripheral blood mononuclear cells (PBMC) were performed using density gradient centrifugation using OptiPrep solution (Sigma-Aldrich, St. Louis, MO) as previously described [52]. In brief, OptiPrep gradient solution was used to separate PBMC and plasma layers at centrifugation at 300 g and 20 °C for 30 min. PBMC were further washed twice with Tricine-buffered saline and collected by centrifugation (150 g at 20 °C for 7 min).

### Quantification of IL1β and IL6

The snap-frozen mouse brain tissues were analyzed for IL1β and IL6 based on our published studies [53, 54]. Briefly, one-half of the hippocampus and amygdala were first homogenized with pellet pestle in RIPA buffer containing protease and phosphatase inhibitors (Thermo Fisher Scientific, Waltham, MA). These were further homogenized by sonication (Mesonix Sonicater 3000) using six pluses of 20 s each on ice. The homogenates were agitated on ice for 30 min and then centrifuged at 14,000 × *g* at 4 °C for 30 min. The supernatant was immediately used to determine the levels of IL1β and IL6 using Quantikine Mouse ELISA kits and following the manufacturer’s protocols (R&D Systems, Minneapolis).

### Immunoblot analysis

Brain tissue homogenates in RIPA buffer containing protease and phosphatase inhibitors were mixed with NuPAGE LDS sample buffer (Thermo Fisher Scientific) and immediately proteins (10 µg) were separated by SDS PAGE electrophoresis (Bio-Rad Laboratories, Hercules, CA) on 4–12% Bis–Tris gradient gels (Thermo Fisher Scientific). The proteins were transferred onto the PVDF membrane (Bio-Rad Laboratories). The membranes were blocked in 5% w/v bovine serum albumin (IgG-free and protease-free BSA, Jackson ImmunoResearch Laboratories, West Grove, PA) in 50 mM Tris–HCl (pH 7.4), 150 mM NaCl and 0.1% Tween 20 (Tris-buffered saline with Tween, TBST). The membranes were incubated at 4 °C overnight with the following mouse-specific primary antibodies: anti-NLRP3 (D4D8T, rabbit monoclonal antibody, 1:1000, #15101, Cell Signaling Technology, Danvers, MA), anti-cleaved Caspase 1 (p20, Asp296, E2G2I, rabbit monoclonal antibody,1: 1000, #89332, Cell Signaling Technology), anti-phospho-Btk (Tyr223, D9T6H, rabbit monoclonal antibody, 1:1000, #8141, Cell Signaling Technology), anti-Btk (D3H5, rabbit monoclonal antibody, 1:1000, #8547, Cell Signaling Technology) and anti-alpha tubulin for loading control (ab4074, rabbit polyclonal antibody, 1:5000, Abcam). The membranes were washed five times with TBST and incubated with horseradish peroxidase-conjugated goat anti-rabbit IgG secondary antibody (adsorbed with mouse and bovine to prevent cross-reactivity, 1:10,000, #STAR124P, Bio-Rad Laboratories) in TBST for 1 h at room temperature. The membranes were washed five times with TBST and immunoreactivity was detected using SuperSignal West Pico PLUS Chemiluminescent substrate (Thermo Fisher Scientific).

### Caspase 1 activity assay

The activation of Caspase 1 in mouse brain samples were determined using Caspase 1 Colorimetric Assay Kit (YVAD, Merck Millipore, Burlington, MA) and following the manufacturer’s instructions. The assay determines the activity of Caspase 1 that recognizes the sequence YVAD. In brief, hippocampus and amygdale tissue samples from one brain hemisphere were homogenized in an ice-cold lysis buffer. The lysates were added to a Caspase 1 reaction buffer in a 96-well flat-bottom microplate. A substrate solution containing YVAD conjugated to chromophore p-nitroanilide (pNA) was added to each well and this was followed by incubation at 37 °C for 2 h. The quantification of Caspase 1 activity was carried out by the detection of pNA from the cleavage of the pNA-YVAD substrate by measuring light emission at 405 nm using a microplate reader (Molecular Devices, San Jose, CA).

### Statistical analysis and reporting of data

All analysis was carried out using the open-source program JASP (JASP Team 2020, Version 0.14). All main effects are reported with eta square (*η*^2^) and partial eta square (*η*_*p*_^2^) for one-way and factorial ANOVA tests, respectively, to provide information about effect sizes for each test. In factorial ANOVAs, if interaction terms were significant, the main effects were not tested post hoc. Instead, interactions were broken down using proper Bonferroni-corrected tests. For each post hoc comparison, the t values are reported alongside p values, rather than Cohen’s d as measures of effect size, since the Cohen’s d is not corrected for multiple comparisons in JASP. In case of 2X2X2 factorial ANOVAs, for each analysis, the results are reported with the significant main effects and the interaction term that had the largest effect size (*η*_*p*_^2^). If, however, the 3-way interaction term was statistically significant, then this was reported and post hoc testing was done accordingly. *P* < 0.05 was considered statistically significant for all analysis. Unless specified, all data reported in the text are reported as mean ± SD. Plots were constructed using GraphPad Prism 8.0 (GraphPad Software, San Diego, CA) and figure layouts created using Adobe Creative Suite and Microsoft Office.

## Results

### Both predator odor stress and physical stress lead to hyper-anxious behavior in mice and elevated neuroinflammatory markers in the brain at 1-week post-stress

We conducted two different stress paradigms, one was psychological stress and another was physical stress, as described in the methods section. Psychological stress was emulated by exposing 14-week-old mice to fresh rat feces for three hours away from home cage. Rats are natural predators for mice and their odor is well known to induce anxiety behavior in mice [55, 56].

To impart physical stress, we used a combination of physical restraint, forced swim and underwater submersion, as discussed in the methods section. We assessed anxious behavior 7 days post-stress and soon after analyzed the animals’ brains for neuroinflammatory markers. To control for stress from the surroundings in the stress experiment arena, we used two different sets of control mice. The first group of control animals were brought into the stress experimental arena to expose them to the surroundings, however, kept away from the fume hood where stress experiments were carried out to avoid the confound of any vicariously acquired stress. The second group of control rodents was kept in the vivarium away from the stress experiment arena.

**Fig. 1:**
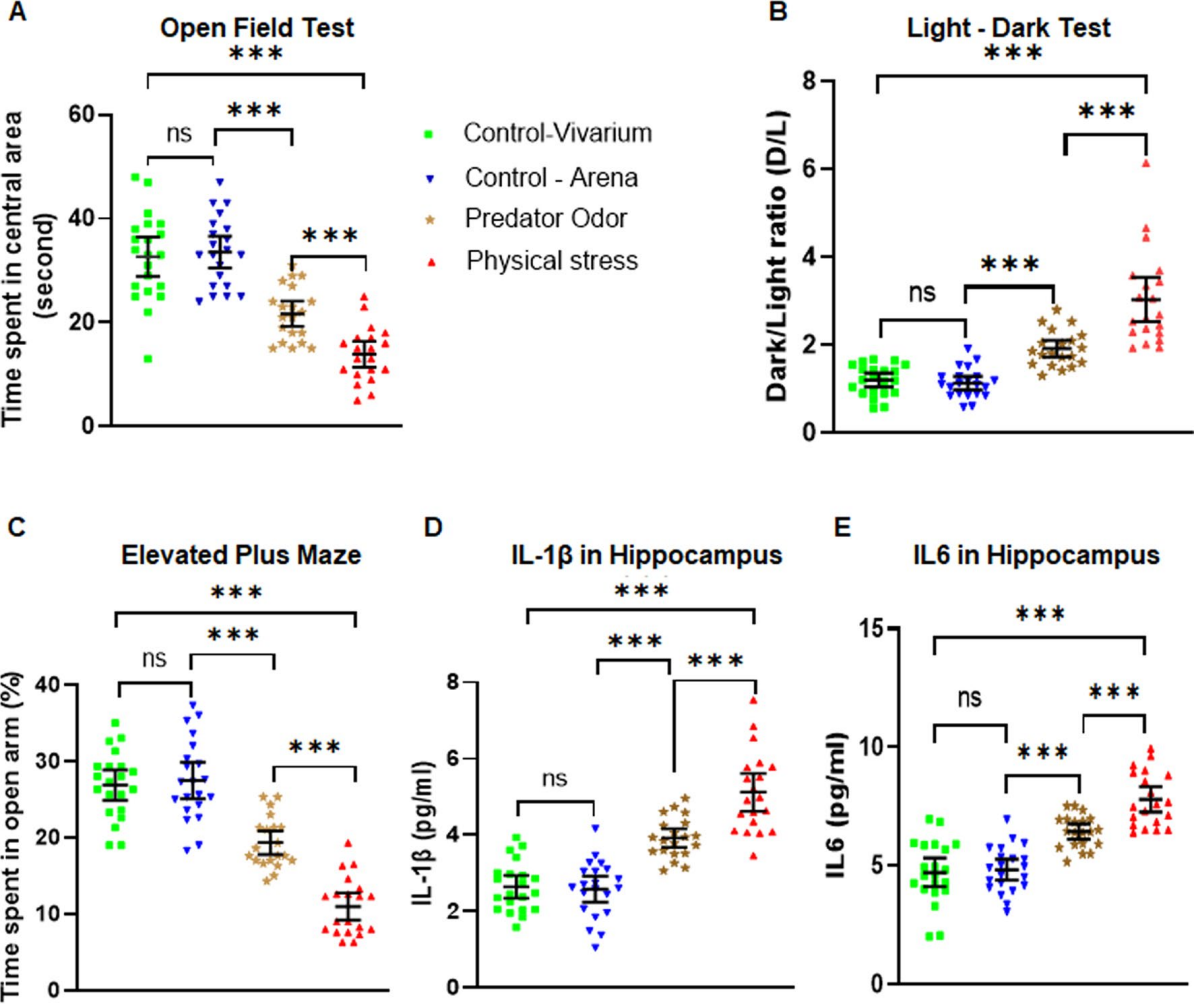
Physical and psychological stress induces hyper-anxious behavior in mice.** A** Examination of anxious behavior of stressed and non-stressed control mice using open field test (OFT). Mice exposed to predator odor and physical stress exhibited significantly higher anxiety levels when compared to the controls (Control-vivarium, and Control-arena), as depicted by their hesitation to explore or spend more time in the central area of the OFT. **B** Light–dark test (LDT) revealed mice exposed to predator odor and restrain stress exhibited significantly higher anxiety levels (dark–light ratios) when compared to the controls, as explained by their reluctance to spend more time in the light chamber of the LDT, with physically stressed mice displaying greater anxious behavior. **C** Elevated plus maze (EPM) test revealed mice subjected to predator odor, and physical stress displayed significantly increased anxiety levels compared to the controls, as evidenced by their hesitation to spend more time in the open arms of the EPM. **D **Examination of Interleukin 1β (IL1β) in the hippocampus of mice exposed to predator odor and physical stress showed aberrantly higher IL1β levels compared to the controls. **E** Examination of Interleukin 6 (IL6) in the hippocampus of mice by ELISA showed that mice exposed to predator odor and physical stress showed aberrantly higher IL6 levels relative to the controls. All data are presented as mean with 95% CI (*n* = 20–21/group); ***p < 0.001, ns (not significant); one-way *ANOVA* followed by Bonferroni post hoc test

Animals exposed to predator odor and subjected to physical stress demonstrated significantly reduced crossings of the central field in an open field test, a behavior that is associated with increased anxiety in rodents (Fig. 1A). A one-way ANOVA revealed a significant effect of the experimental stress manipulations [*F* *(3,78)* = 42.16, *p* < 0.001, η^*2*^ = 0.619; Fig. 1A]. Pairwise comparisons with the Bonferroni correction demonstrated that the mice in the physical stress group performed worst (mean ± SD: 13.9 ± 5.24), followed by the mice exposed to predator odor (21.7 ± 5.18), compared to both sets of control mice (*p* < 0.001). The control mice from vivarium (32.67 ± 8.38) or in the stress arena with no direct view of the stress paradigms (33.57 ± 6.74) were not significantly different from one another in the time spent in the central square of the field (*p* > 0.05).

The second behavior test to access the anxiety in mice was the light–dark test. Naïve mice spend roughly equivalent amount of time exploring both the light and the dark compartments of the testing chambers with a slight preference on the dark compartment. A tendency of spending more time in the dark half of the testing chamber is associated with increased anxiety in mice. The ratio of the time spent by the mice in the dark to the time spent in the lighted half of the testing chamber (D/L ratio) should therefore be approximately 1 or slightly above 1 in control mice, and an increase in this ratio would reflect increased anxious behavior in mice. A one-way ANOVA of the experimental groups revealed a significant difference in group means by experimental status [*F* *(3,78)* = 41.54, *p* < 0.001, *η*^2^ = 0.615; Fig. 1B]. Bonferroni-corrected post hoc tests were used for pairwise comparisons. While both the control groups of mice, i.e., one in the vivarium and another exposed to the stress experiment arena displayed similar D/L ratios (1.19 ± 0.34 and 1.13 ± 0.36; *t* = 0.37, *p* < 0.05), mice exposed to predator odor exhibit a significant increase in the D/L ratio (1.92 ± 0.41) compared to control mice in the arena (*t* = 4.06, *p* < 0.001) and control mice in the vivarium (*t* = 3.69, *p* < 0.001). Mice subjected to physical stress exhibited the highest D/L ratio (3.03 ± 1.08), significantly more compared to mice exposed to predator odor, (*t* = 5.72, *p* < 0.001), control mice in the arena (*t* = 9.85, *p* < 0.001), and control mice in the vivarium (*t* = 9.49, *p* < 0.001).

To further evaluate the anxiety behavior in stressed mice, we conducted the well-established elevated plus maze (EPM) test. Time spent in the open arm of the maze was used to measure anxious behavior, with a reduction in the time spent in the open arm taken as a manifestation of increased anxiety. A one-way ANOVA revealed a significant difference among group means [*F* *(3,78)* = 67.13, *p* < 0.001, *η*^2^ = 0.721; Fig. 1C]. Post hoc testing with the Bonferroni correction was used for pairwise comparisons. Mice subjected to physical stress spent the shortest time in the open arm of the maze in seconds (33.05 ± 11.35), compared to both groups of control mice in the stress experiment arena (82.54 ± 15.01; *t* = 12.38, *p* < 0.001) and in the vivarium (80.71 ± 13.07; *t* = 11.93, *p* < 0.001), as well as the mice exposed to predator odor (58.1 ± 9.98; *t* = 6.2, *p* < 0.001). Mice exposed to predator odor also displayed significantly higher anxiety manifested by the shorter times they spent in the open arm of elevated maze, as compared to the control mice near the arena (*t* = 6.114, *p* < 0.001) and the vivarium (*t* = 5.661, *p* < 0.001). The two control groups were not significantly different from one another in this regard (*t* = 0.5, *p* = 1.0).

The above experiments demonstrated that the stress paradigms we conducted on mice elicited a robust anxiety response, and predictably, a higher response in mice subjected to physical stress. Next, we focused on the neuroinflammatory markers of the brain, specifically the proinflammatory cytokines IL1β and IL6 in the hippocampus. An ELISA assay for IL1β was conducted and analyzed by one-way ANOVA. Both physical stress and predator odor stress elicited a robust IL1β upregulation in the hippocampus of the mice, compared to control hippocampi [*F* *(3,78)* = 49. 99, *p* < 0.001, *η*^2^ = 0.658; Fig. 1D]. Post hoc analysis (Bonferroni corrected) revealed that physical stress led to the most robust upregulation of IL1β (pg/ml) in the hippocampus (5.12 ± 1.06), compared to both groups of control mice in the stress experimental arena (2.58 ± 0.75; *t* = 10.54, *p* < 0.001) and in the vivarium (2.64 ± 0.65; *t* = 10.29, *p* < 0.001), as well as the mice exposed to predator odor (3.92 ± 0.53; *t* = 4.92, *p* < 0.001). IL1β levels in mice exposed to predator odor were significantly higher as compared to the control mice near the arena (*t* = 5.59, *p* < 0.001) and the vivarium (*t* = 5.3, *p* < 0.001). IL1β levels were similar between the two control groups (*t* = 0.25, *p* = 1.0).

IL6 levels in the hippocampus followed a very similar pattern to that of IL1β. A one-way ANOVA revealed significant differences in group means based on experimental conditions [*F* *(3,78)* = 43. 79, *p* < 0.001, *η*^2^ = 0.599; Fig. 1E]. Physical stress upregulated IL6 most robustly (7.79 pg/ml ± 1.15), compared to both groups of control mice in the arena (4.82 pg/ml ± 0.97; *t* = 8.95, *p* < 0.001) and in the vivarium (4.71 pg/ml ± 1.32; *t* = 9.29, *p* < 0.001), as well as the mice exposed to predator odor (6.43 pg/ml ± 0.69; *t* = 4.06, *p* < 0.001).M ice exposed to predator odor displayed significantly elevated IL-6 compared to the control mice near the arena (*t* = 4.84, *p* < 0.001) and the vivarium (*t* = 5.18, *p* < 0.001). The two control groups were not significantly different from one another in their IL6 expression levels in the hippocampus (*t* = 0.34, *p* = 1.0).

Taken together, the results of the experiment suggested that alongside anxious behavior, our stress models also upregulated proinflammatory cytokines IL1β and IL6 in the brain. Physical stress elicited a more prominent response across all behavioral as well as molecular measures. Since the two control groups had no significant difference in any of the outcome measures, for the rest of this study, we conducted our experiments with control mice which were exposed to the stress experiment arena without visual access to the area where stress paradigms were performed.

### Female mice display an exacerbated stress response compared to male mice

To understand the gender differences in anxiogenic response, we examined the male and female mice under both stress paradigms, i.e., psychological stress (predator odor) and physical stress (restraint stress followed by underwater trauma). We observed that female mice had a more pronounced response to stress in both the stress models used, across behavioral and molecular outcome measures. However, like observed in the earlier experiments, physical stress continued to elicit a more robust response in all outcomes assessed compared to predator odor stress. The sex difference is of interest and special relevance because this mirrors human data on PTSD and stress and anxiety related disorders—females demonstrate a higher incidence and prevalence and severity of anxious symptoms in these disorders [57].

**Fig. 2:**
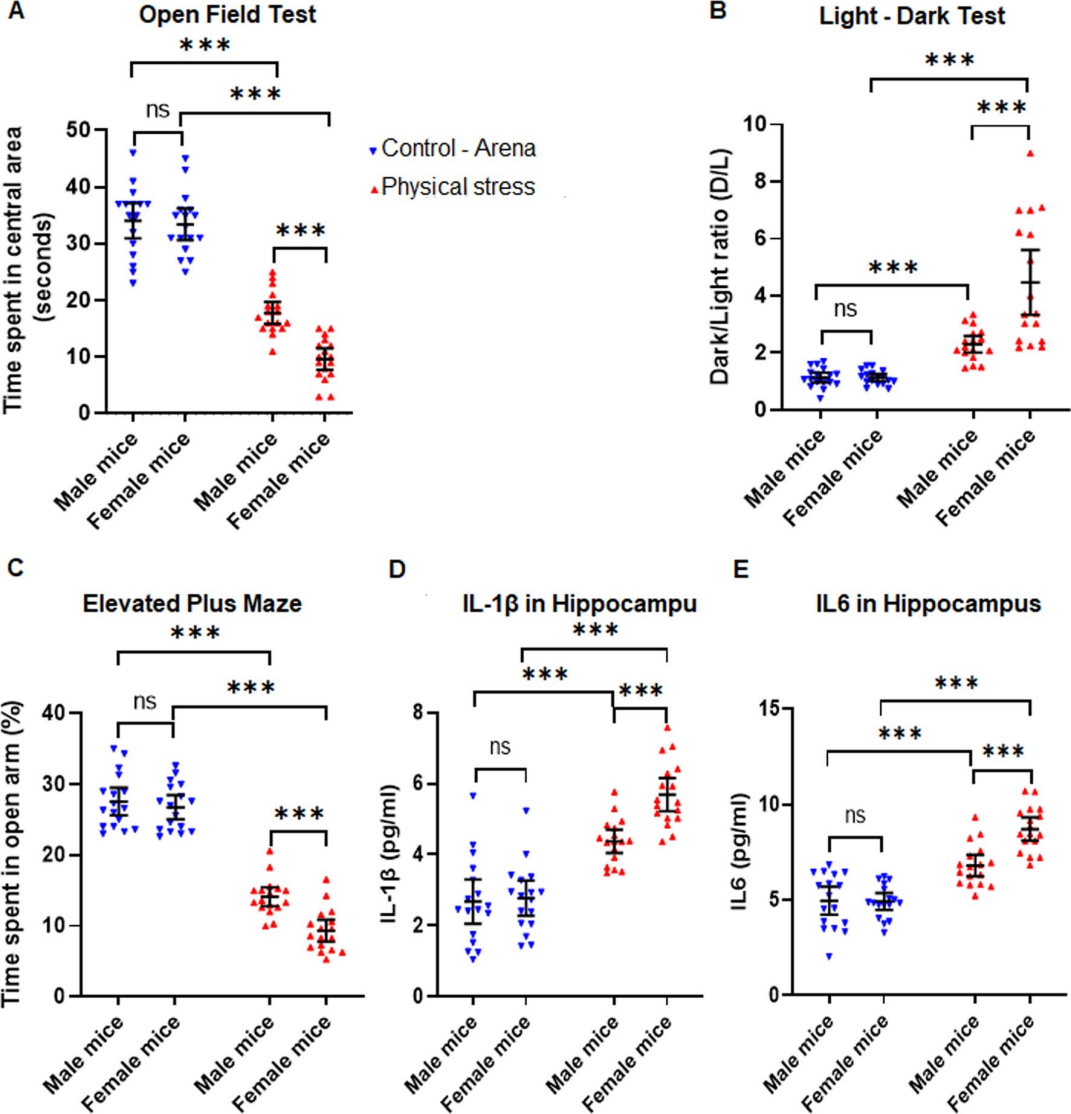
Female mice display an exacerbated stress response compared to male mice.** A** Open field test showing physically stressed mice exhibited significantly higher anxiety levels when compared to the controls, as illustrated by their reluctance to explore or spend more time in the central area of the OFT, with the female stressed mice showing greater anxiety. **B** Dark–Light test revealed female mice exposed to physical stress exhibited significantly higher anxiety levels (higher dark/light ratios) when compared to other groups, as explained by their hesitation to spend more time in the light chamber of the LDT. **C** Elevated plus maze revealed female mice subjected to physical stress displayed significantly increased anxiety levels compared to those in stressed males, as evidenced by their hesitation to spend more time in the open arms of the EPM. **D** Interleukin 1β (IL1β) levels in the hippocampus revealed stressed female mice showed aberrantly higher IL1β activity compared to those in stressed male mice. **E** Female mice subjected to physical stress showed aberrantly higher IL6 levels in the hippocampus relative to their male counterparts. All data are presented as mean with 95% CI (*n* = 17/group); ***p < 0.001, ns (not significant); two-way ANOVA followed by Bonferroni post hoc test

Physically stressed mice, both male and female were assessed by the open field test (Fig. 2A), the light–dark test (Fig. 2B) and the elevated plus maze test (Fig. 2C). We conducted separate analysis between groups using factorial ANOVAs for each test, with stress (Control mice near the arena vs. physically stressed mice) and sex (male vs. female) as factors. For the open field test, there was a significant main effect of stress [*F* *(1,64)* = 294.76, *p* < 0.001, *η*_*p*_^2^ = 0.82] and a significant main effect of sex [*F* *(1,64)* = 13.97, *p* < 0.001, *η*_*p*_^2^ = 0.17] on the time spent in the central square of the open field. This was further qualified by a significant interaction between the two factors [*F* (*1,64*) = 10.14, *p* = 0.002, *η*_*p*_^2^ = 0.14; Fig. 2A]. Bonferroni-corrected post hoc testing revealed that while control male and female mice spent similar times in the central square of the open field (M: 34.06 s ± 6.05, F: 33.41 s ± 5.39; *t* = 0.39, *p* = 1.00), among physically stressed mice, the time spent by female mice (9.65 s ± 3.71) in the central square of the open field test was significantly less than the male mice (17.77 s ± 3.70; *t* = 4.90, *p* < 0.001). Between the different experimental conditions, control male mice spent a significantly higher time in the central square compared to the physically stressed male mice (*t* = 9.83, *p* < 0.001). Likewise control female mice spent a much higher time in the central square compared to physically stressed female mice (*t* = 14.33, *p* < 0.001).

In the light–dark test, analysis between subjects using factorial ANOVA on the D/L ratio revealed a significant main effect of stress [*F* (*1,64*) = 64.51, *p* < 0.001, *η*_*p*_^2^ = 0.50], a significant main effect of sex [*F* (*1,64*) = 15.2, *p* < 0.001, *η*_*p*_^2^ = 0.19] and a significant interaction between stress and sex [[*F* (*1,64*) = 15.00, *p* < 0.001, *η*_*p*_^2^ = 0.19; Fig. 2B]. Bonferroni post hoc analysis showed that while control male and female mice had similar D/L ratios (M: 1.13 ± 0.34, F: 1.14 ± 0.24; *t* = 0.001, *p* = 1.00), physically stressed female mice had significantly higher D/L ratios compared to physically stressed male mice (M: 2.3 ± 0.56, F: 4.47 ± 2.19; *t* = 5.48, *p* < 0.001). Control male mice exhibited a significantly lower D/L ratio than physically stressed male mice (*t* = 2.94, *p* = 0.027), as did control female mice compared to physically stressed female mice (*t* = 8.42, *p* < 0.001).

Results from the elevated plus maze (time spent in the open arm of the maze) was also compared fully between-subjects using factorial ANOVA, and demonstrated a significant main effect of stress [*F* (*1,64*) = 390.60, *p* < 0.001, *η*_*p*_^2^ = 0.86], a significant main effect of sex, [F (*1,64*) = 12.85, *p* < 0.001, *η*_*p*_^2^ = 0.17], and a significant interaction between stress and sex, [*F* (*1,64*) = 6.519, *p* = 0.013, *η*_*p*_^2^ = 0.09; Fig. 2C]. Once again, control male and female mice exhibited a similar amount of time spent in the open arm (M: 27.56 s ± 3.295, F: 26.76 s ± 3.82; *t* = 0.88, *p* = 1.00), but physically stressed female mice spent a significantly lower time in the open arm than physically stressed male mice (M: 14.12 s ± 2.61, F: 9.33 s ± 3.03; *t* = 4.34, *p* < 0.001). Within the different experimental conditions, the control male mice spent a significantly higher time in the open arm compared to the physically stressed mice (*t* = 12.17, *p* < 0.001) as did the female control mice compared to the physically stressed female mice (*t* = 15.78, *p* < 0.001).

Thus far, in all three behavior assessments, stressed female mice displayed a hyper-anxious behavior. Next, we wanted to investigate proinflammatory cytokines in the brain and see if they were consistent with the exaggerated anxiety response in the mice. To this end, we performed ELISA assays to test for hippocampal IL1β and IL6 in this cohort. In each case, we analyzed the results with individual 2(arena control, physical stress) X 2(male, female) factorial ANOVAs followed with Bonferroni-corrected pairwise comparisons for the significant main effects and interactions. For hippocampal IL1β, the factorial ANOVA revealed an extremely significant main effect of stress, [*F* (*1,64*) = 99.24, *p* < 0.001, *η*_*p*_^2^ = 0.61], a significant main effect of sex [*F* (*1,64*) = 9.32, *p* = 0.003, *η*_*p*_^2^ = 0.13] and a small yet significant interaction between stress and sex [*F* (*1,64*) = 9.32, *p* = 0.011, *η*_*p*_^2^ = 0.09; Fig. 2D]. Consistent with the behavioral data, the stressed female mice had a significantly higher level of IL1β compared to the stressed male mice (M: 4.37 pg/ml ± 0.64, F: 5.69 pg/ml ± 0.91; *t* = 4.02, *p* < 0.001), while control male and female mice had similar levels of hippocampal IL1β (M: 2.68 pg/ml ± 1.21, F: 2.78 pg/ml ± 0.96; *t* = 0.29, *p* = 1.00). Within the different experimental conditions, physically stressed female mice had much higher levels than control female mice (*t* = 8.91, *p* < 0.001) and a similar pattern of difference was also observed between control males and stressed males, although the magnitude of the difference was less between control and stressed groups in the males (*t* = 5.18, *p* < 0.001).

For IL6, the factorial ANOVA revealed an extremely significant main effect of stress [*F* (*1,64*) = 100.79, *p* < 0.001, *η*_*p*_^2^ = 0.61], a significant effect of sex [F (*1,64*) = 11.074, p = 0.001, η_p_^2^ = 0.15] and a significant interaction [*F* (*1,64*) = 11.93, *p* < 0.001, *η*_*p*_^2^ = 0.16; Fig. 2E]. Just like IL1β levels, hippocampal IL6 levels (pg/ml) were higher in stressed females compared to stressed males (M: 6.81 ± 1.10, F: 8.71 ± 1.17; *t* = 4.80, *p* < 0.001) but not in control males versus females (M: 4.96 ± 1.43, F: 4.93 ± 0.86; *t* = 0.09, *p* = 1.00). Stressed male and well as female mice displayed much higher IL6 in the hippocampus than control males and females (*t* = 4.66, *p* < 0.001; *t* = 9.451, *p* < 0.001, respectively). Therefore, we discovered a pattern of proinflammatory cytokine expression that mirrors the sex difference seen in the behavioral data following physical stress, compared to control animals. We repeated these experiments with animals exposed to predator odor stress and observed similar anxiety responses with the three above-mentioned behavioral tests as well as elevations in hippocampal IL1β and IL6, as measured with ELISA (Additional file 1: Fig. S1A–E). The sex differences were apparent in that cohort as well, however, the magnitude of the effects was lesser than what was elicited by the physical stress model. Keeping in mind the robust responses we saw following physical stress, hereafter, for all mechanistic investigation, we used the physical stress model and mice near stress experiment arena without a direct line of sight to the stress experiments as controls.

### Signaling pathways that lead to production of IL1β are upregulated in the hippocampus and amygdala of physically stressed female mice to a greater extent than male mice

Next, we focused on molecules that are involved in the production of IL1β in two brain areas which always figure prominently in the literature related to stress and anxiety—hippocampus and amygdala. Using immunoblot, we probed these brain areas in physically stressed mice as well as control mice at one-week post-stress. We observed a robust induction of the NLRP3 inflammasome, as well as cleaved Caspase 1 (p20), the key molecules responsible for the generation of IL1β.

**Fig. 3:**
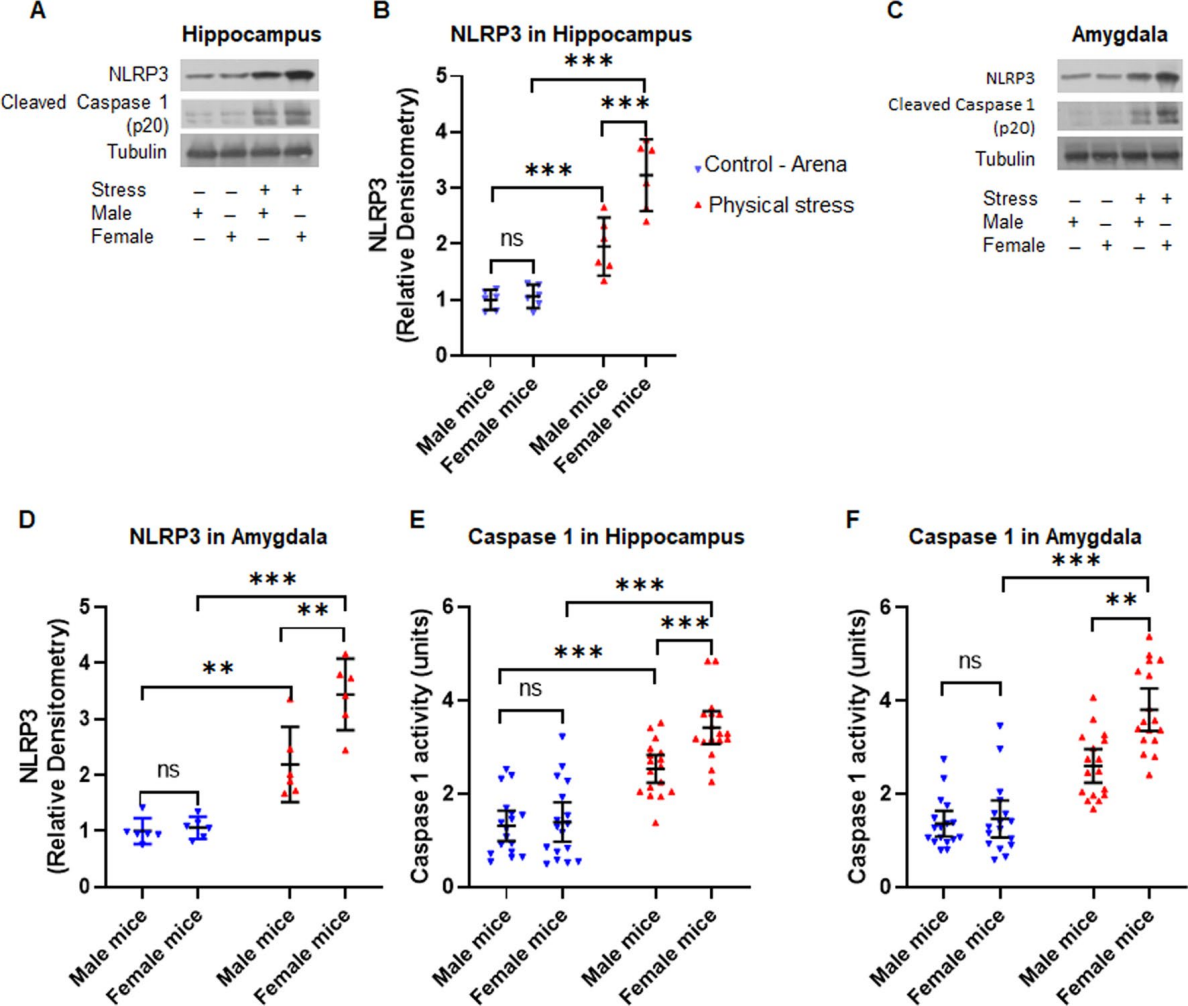
Physical stress leads to induction of NLRP3 inflammasome and Caspase 1 activation in hippocampus and amygdala of mice. **A** Immunoblot analysis of NLRP3 and cleaved Caspase 1 from hippocampus homogenates. **B** The relative densitometry of NLRP3 immunoblots of samples from the hippocampus, which shows physical stress-induced higher levels of NLRP3 inflammasome in female mice in comparison to male mice**. C** Western blot analysis of NLRP3 and cleaved Caspase 1 in amygdala homogenates. **D** The relative densitometry analysis of NLRP3 immunoblots of amygdala samples. The analysis showed stressed female mice exhibited increased induction of NLRP3 inflammasome in comparison to male mice. Caspase 1 activity in the hippocampus **E** and amygdala (**F**), as measured in respective brain homogenates using Caspase 1 assay kit. Female mice subjected to physical stress showed significantly higher Caspase 1 activation relative to their male counterparts and control mice. All values are presented as mean with 95% CI (*n* = 6–17/group); ***p < 0.001, ns (not significant); two-way ANOVA followed by Bonferroni post hoc test

Consistent with the results shown in Fig. 2, the induction NLRP3 and cleaved Caspase 1 (p20) were more prominent in stressed female mice, in comparison with stressed male mice and control group of mice, in both the hippocampus (Fig. 3A) and the amygdala (Fig. 3C). We normalized the NLRP3 immunoblot band intensities against tubulin and compared the relative band intensities from all groups with 2(control, physical stress) X 2 (male, female) factorial ANOVAs for hippocampus and amygdala each. For samples from hippocampus, the factorial ANOVA demonstrated a significant main effect for stress[*F* (*1,20*) = 84.89, *p* < 0.001, *η*_*p*_^2^ = 0.81], a significant main effect of sex [*F* (*1,20*) = 15.60, *p* < 0.001, *η*_*p*_^2^ = 0.44] on relative band intensities for NLRP3, qualified by significant interaction between stress and sex [*F* (*1,20*) = 12.90, *p* = 0.002, *η*_*p*_^2^ = 0.39; Fig. 3B]. The stressed female mice had a significantly higher relative NLRP3 band intensity compared to the stressed male mice (M: 1.95 ± 0.5, F: 3.23 ± 0.61; *t* = 5.33, *p* < 0.001), while control male and female mice had similar levels of hippocampal NLRP3 (M: 1.0 ± 0.17, F: 1.06 ± 0.2; *t* = 0.25, *p* = 1.00). Within the different experimental conditions, physically stressed female mice had much higher NLRP3 levels than control female mice (*t* = 9. 4, *p* < 0.001) and a similar pattern of difference was also observed between control males and stressed males, although the magnitude of the difference was less between control and stressed groups in the males (*t* = 3.96, *p* = 0.005).

The relative band intensities of immunoblot from amygdala samples also followed a similar pattern (Fig. 3D). The factorial ANOVA demonstrated a significant main effect for stress [*F* (*1,20*) = 84.74, *p* < 0.001, *η*_*p*_^2^ = 0.82], a significant main effect of sex [*F* (*1,20*) = 11.91, *p* < 0.001, *η*_*p*_^2^ = 0.37] on relative band intensities for NLRP3 from amygdala, qualified by significant interaction between stress and sex [*F* (*1,20*) = 9.87, *p* = 0.005, *η*_*p*_^2^ = 0.33; Fig. 3D]. Once again, the amygdala samples from stressed female mice had a significantly higher relative NLRP3 intensity as compared to the stressed male mice (M: 2.19 ± 0.64, F: 3.44 ± 0.61; *t* = 4.66, *p* < 0.001). Within the different experimental conditions, physically stressed female mice had significantly higher NLRP3 levels in amygdala than control female mice (*t* = 8.88, *p* < 0.001) and a similar pattern of difference was also observed between control males and stressed males, although the magnitude of the difference was less between control and stressed groups in the males (*t* = 4.44, *p* = 0.002).We also observed similar pattern from analysis of Caspase 1 activity in hippocampus (Fig. 3E) and amygdala (Fig. 3F). The physically stressed female mice had higher Caspase 1 activity as compared to their stressed male counterparts and controls. Similarly, the quantification of cleaved Caspase 1 relative band intensities from immunoblots showed significantly higher band intensities in physically stressed females as compared to stressed males and control groups in both amygdala (Additional file 1: Fig. S2A) and hippocampus (Additional file 1: Fig. S2B).

NLRP3 inflammasome play a critical role in the pathophysiology of neuroinflammation through activation of Caspase 1 and IL1β cytokine. Our data until here suggest that physical stress elicits hyper-anxious behavior, robust NLRP3 induction in the hippocampus and amygdala, alongside activation of downstream mediators IL1β and Caspase 1, and all responses are more pronounced in females compared to males.

### Pharmacological inhibition of the NLRP3 inflammasome with MCC950 attenuates sexually divergent anxious behavior in mice as well as IL1β and Caspase 1 activity in the hippocampus and amygdala

Next, we carried out a pharmacological NLRP3 inhibition study, which served as proof-of-concept experiments to verify whether targeting NLRP3 pharmacologically can suppress the IL1β production and rescue the anxious behavior in mice subjected to restrained stress and underwater trauma. To this end, we selected MCC950, a compound known to inhibit NLRP3 and have an excellent blood–brain barrier permeability [34, 58]. We injected control as well as physically stressed mice with vehicle and MCC950 (50 mg/kg, i.p.) and tested the mice in the three behavioral paradigms mentioned before as well as analyzed their brains biochemically for IL1β and related markers. For each outcome measure we used 2(control, stressed) X 2(male, female) X 2 (vehicle, MCC950) Factorial ANOVAs. Given the pattern of interactions noted in the earlier set of experiments, we hypothesized a priori that we will see significant interactions among the factors, and the patterns of the interactions were especially of interest to us. We also wanted to see if the drug by itself influenced behavior and neuroinflammatory correlates in control mice, in addition to the drug’s ability to rescue phenotypes in stressed mice, as well as gender divergent effects.

**Fig. 4:**
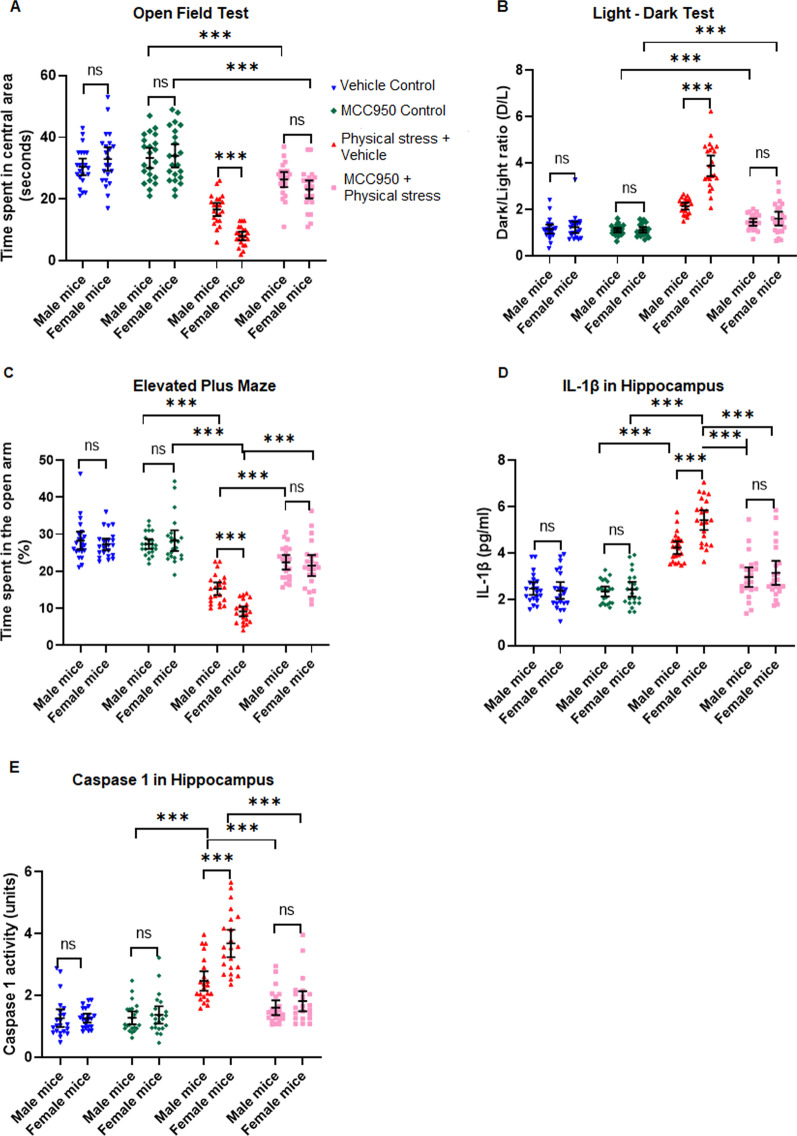
Inhibition of NLRP3 inflammasome by MCC950 treatment attenuates anxious behavior and neuroinflammation in stressed mice. Physical stress was induced by subjecting mice to restraint and underwater trauma. Control mice were treated with either vehicle or MCC950. Similarly, stressed mice were treated with either vehicle or MCC950. **A** Open field test: as compared to controls, physically stressed mice dosed with MCC950 exhibited significantly decreased anxiety levels, as illustrated by the increased time spent in the central area of the OFT, in both male and female mice. **B** Light–dark test: physically stressed mice dosed with MCC950 exhibited significant improvement from hyper-anxious behavior, as explained by the reduced D/L ratio, i.e., increased exploration time in the light chamber of the LDT. **C** Elevated plus maze test revealed physical stress mice dosed with MCC950 displayed significant rescue from anxiety compared to the vehicle controls, as evidenced by the increased duration of time spent in the open arms of the EPM. **D** Examination of IL1β in hippocampal homogenates by ELISA revealed physically stressed mice dosed with MCC950 showed diminished IL1β levels compared to the vehicle controls, denoting significant rescue from anxiety. **E **Caspase 1 activity in the hippocampus: physically stressed mice administered with MCC950 exhibited attenuated Caspase 1 activation relative to their vehicle controls. All values are presented as mean with 95% CI (n = 22/group); ***p < 0.001, ns (not significant); 2X2X2 factorial ANOVA followed by Bonferroni post hoc test

When tested on the open field with the time spent in the central square of OFT and analyzed by a factorial ANOVA (Fig. 4A), there was a significant main effect of stress [*F* (*1,168*) = 205.44, *p* < 0.001, *η*_*p*_^2^ = 0.55], a main effect of sex [*F* (*1,168*) = 4.68, *p* = 0.032, *η*_*p*_^2^ = 0.03], and a significant main effect of drug [*F* (*1,168*) = 52.86, *p* < 0.001, *η*_*p*_^2^ = 0.23]. Two interaction terms were significant, namely stress*sex [*F* (*1,168*) = 14.41, *p* < 0.001, *η*_*p*_^2^ = 0.08] and stress*drug [*F* (*1,168*) = 27.75, *p* < 0.001, *η*_*p*_^2^ = 0.14]. Control mice both vehicle treated (M: 30.35 s ± 6.04, F: 32.95 s ± 8.69; *t* = 0.66, *p* = 1.00) or MCC950 treated (M: 33.31 s ± 7.31, F: 34.05 s ± 8.48; *t* = 0.39, *p* = 1.00) did not exhibit significant difference within sex or treatment (M: *t* = 0.492, *p* = 0.95). Vehicle-treated mice subjected to physical stress, however, displayed a sharp decline in the time spent in the central square of the open field which with the decline being sharper in female mice than males (M: 16.64 s ± 4.81, F: 8.05 s ± 3.14) compared to control animals, both vehicle treated (*t* = 13.86, *p* < 0.001) as well as MCC950 treated (*t* = 13.86, *p* < 0.001). Physically stressed mice dosed with MCC950 showed a significant rescue from vehicle-treated stressed mice (M: 26.32 s ± 5.32, F: 23.14 s ± 6.52) compared to vehicle-treated stressed mice (*t* = 8.87, *p* < 0.001).

The light–dark test showed a similar pattern (Fig. 4B). The factorial ANOVA conducted on the D/L ratios revealed a significant main effect of stress [*F* (*1,168*) = 191.405, *p* < 0.001, *η*_*p*_^2^ = 0.53], a main effect of sex [*F* (*1,168*) = 36.97, *p* < 0.001, *η*_*p*_^2^ = 0.18], and a significant main effect of drug [*F* (*1,168*) = 93.43, *p* < 0.001, η_p_^2^ = 0.36]. This was further qualified by significant interaction terms, including a 3-way interaction between stress, sex and drug [*F* (*1,168*) = 22.058, *p* < 0.001, *η*_*p*_^2^ = 0.12]. Control mice both vehicle treated (M: 1.15 ± 0.45, F: 1.22 ± 0.55; *t* = 0.02, *p* = 1.00) or MCC950 treated (M: 1.1 ± 0.22, F: 1.11 ± 0.26; *t* = 0.98, *p* = 1.00) did not exhibit significant difference within sex or drug treatment (males, *t* = 0.29, p = 1.00; Females, *t* = 0.71, *p* = 1.00). Vehicle-treated mice subjected to physical stress, however, showed a markedly increased D/L ratio, with female ratios being significantly higher (M: 2.141 ± 0.34, F: 3.89 ± 0.99; *t* = 10.75, *p* < 0.001) compared to control animals, both vehicle treated (M: *t* = 6.01, *p* < 0.001; F: *t* = 16.4, *p* < 0.001) as well as MCC950 treated (M: *t* = 6.39, p < 0.001; M: *t* = 17.11, *p* < 0.001). The D/L ratio was restored in physically stressed mice treated with MCC950 and showed a significant rescue (M: 1.45 ± 35, F: 1.6 ± 0.68, *t* = 0.93, *p* = 1.00) and their performance was not significantly different from MCC950 treated control mice (M: *t* = 0.4, *p* = 0.957; F: *t* = 3.04, *p* = 0.078).

Next, we analyzed the data from elevated plus maze (Fig. 4C). We used the time spent by the mice from all groups, in the open arms, and calculated percentage time spent in the open arm. The factorial ANOVA conducted on the EPM data revealed a significant main effect of stress [*F* (*1,168*) = 230.08, *p* < 0.001, *η*_*p*_^2^ = 0.58], a main effect of sex [*F* (*1,168*) = 6.38, *p* = 0.012, *η*_*p*_^2^ = 0.04], and a significant main effect of drug [*F* (*1,168*) = 48.32, *p* < 0.001, *η*_*p*_^2^ = 0.22]. This was further qualified by significant interaction terms, with the most significant interaction between stress and drug [*F* (*1,168*) = 47.29, *p* < 0.001, *η*_*p*_^2^ = 0.22]. Control unstressed mice, both vehicle treated (M: 28.26 ± 5.47, F: 27.24 ± 3.59; *t* = 0.72, *p* = 1.00) or MCC950 treated (M: 27.35 ± 3.1, F: 28.36 ± 6.29; *t* = 0.65, *p* = 1.00) spent comparable times in the open arms and did not exhibit significant difference within sex or drug treatment (*t* = 0.52, *p* = 1.00). Vehicle-treated mice subjected to physical stress, however, showed a markedly reduced percentage time spent in the open arm, with females spending significantly lesser time in the open arm of the EPM (M: 15.27 ± 3.9, F: 9.12 ± 2.96; *t* = 6.15, *p* < 0.001) compared to control animals, both vehicle treated (*t* = 15.59, *p* < 0.001) as well as MCC950 treated (M: *t* = 15.64, *p* < 0.001). The percentage time spent in the open arm was markedly improved in the physically stressed mice treated with MCC950 (M: 22.39 ± 4.4, F: 21.51 ± 6.37, *t* = 0.62, *p* = 1.00).

Thus far, the behavioral data replicated our earlier findings from Fig. 2 with respect to all three behavioral paradigms used to measure anxious behavior. Our model of physical stress produced robust anxious behavior in mice, which was significantly more pronounced in the female mice. When we inhibited the NLRP3 inflammasome, an important upstream molecule that mediates the activation of Caspase 1 and IL1β, using pharmacological inhibitor MCC950, mice showed significant rescue from heightened anxious behavior in all three behavior tests. Thus, it implicates NLRP3–Caspase 1–IL1β pathway in anxiogenesis following physical stress.

We then turned to investigating biochemical changes in the brain following NLRP3 inhibition by administering MCC950. We measured hippocampal IL1β levels with ELISA and analyzed the data with a factorial ANOVA (Fig. 4D). The factorial ANOVA conducted revealed a significant main effect of stress [*F* (*1,168*) = 153.65, *p* < 0.001, *η*_*p*_^2^ = 0.48], a main effect of sex [*F* (*1,168*) = 7.77, *p* = 0.006, *η*_*p*_^2^ = 0.04], and a significant main effect of drug [F (*1,168*) = 58.62, p < 0.001, η_p_^2^ = 0.24]. This was further qualified by significant interaction terms, with the most significant interaction between stress and drug [*F* (*1,168*) = 49.24, *p* < 0.001, *η*_*p*_^2^ = 0.23]. Control mice both vehicle treated (M: 2.47 pg/ml ± 0.62, F: 2.39 pg/ml ± 0.82; *t* = 0.35, *p* = 1.00) or MCC950 treated (M: 2.34 pg/ml ± 0.48, F: 2.44 pg/ml ± 0.72; *t* = 0.65, *p* = 1.00) had similar levels of IL1β in the hippocampus, did not show a sex difference or an upregulation in IL1β production just by the drug treatment (M: *t* = 0.52, *p* = 1.00; F:*t* = 0.22, p = 1.00). Vehicle-treated mice subjected to physical stress, had significantly elevated hippocampal IL1β levels, with more prominent increase in females (M: 4.24 pg/ml ± 0.62, F: 5.42 pg/ml ± 0.62; *t* = 4.79, *p* < 0.001) compared to control animals, both vehicle treated (M: *t* = 7.14, *p* < 0.001; F: *t* = 12.27, *p* < 0.001) as well as MCC950 treated (M: *t* = 7.66, *p* < 0.001; F: *t* = 12.06, *p* < 0.001). When dosed with MCC950, IL-1β levels went down significantly, and abolished the difference between males and females, although females tended to have slightly elevated IL-1β even in treated animals (M: 2.97 pg/ml ± 0.96, F: 3.15 pg/ml ± 1.16, *t* = 0.74, *p* = 1.00). Following MCC950 treatment, hippocampal IL1β levels in physically stressed animals were not significantly higher than vehicle treated (M: *t* = 1.99, p = 1.00; F: *t* = 3.08, p = 0.07) or MCC950 treated (M: *t* = 2.52, *p* = 0.36; F: *t* = 2.86, *p* = 0.13) control mice.

We also analyzed the activity of Caspase 1 in the hippocampus, to validate our results in this pharmacological NLRP3 inhibition experiment, and analyzed the data with a Factorial ANOVA. Overall, the pattern of Caspase 1 activity data (Fig. 4E) mirrored the IL1β data closely. Caspase 1 activity rose sharply and significantly in stressed mice treated with vehicle, and was significantly attenuated in physically stressed mice treated with NLRP3 inhibitor MCC950. We confirmed similar patterns of inhibition of IL1β as well as Caspase 1 activity in the amygdala of stressed mice treated with MCC950 (Additional file 1: Fig. S3 A, B). Therefore, this experiment provided evidence that the NLRP3 inflammasome activation is a crucial part of the neuroinflammatory response seen in this model of physical stress and anxiogenic behavior, with females displaying a more severe phenotype compared to males.

### Bruton’s tyrosine kinase (BTK) is induced in hippocampus and amygdala of mice following physical stress in a sexually divergent pattern

**Fig. 5:**
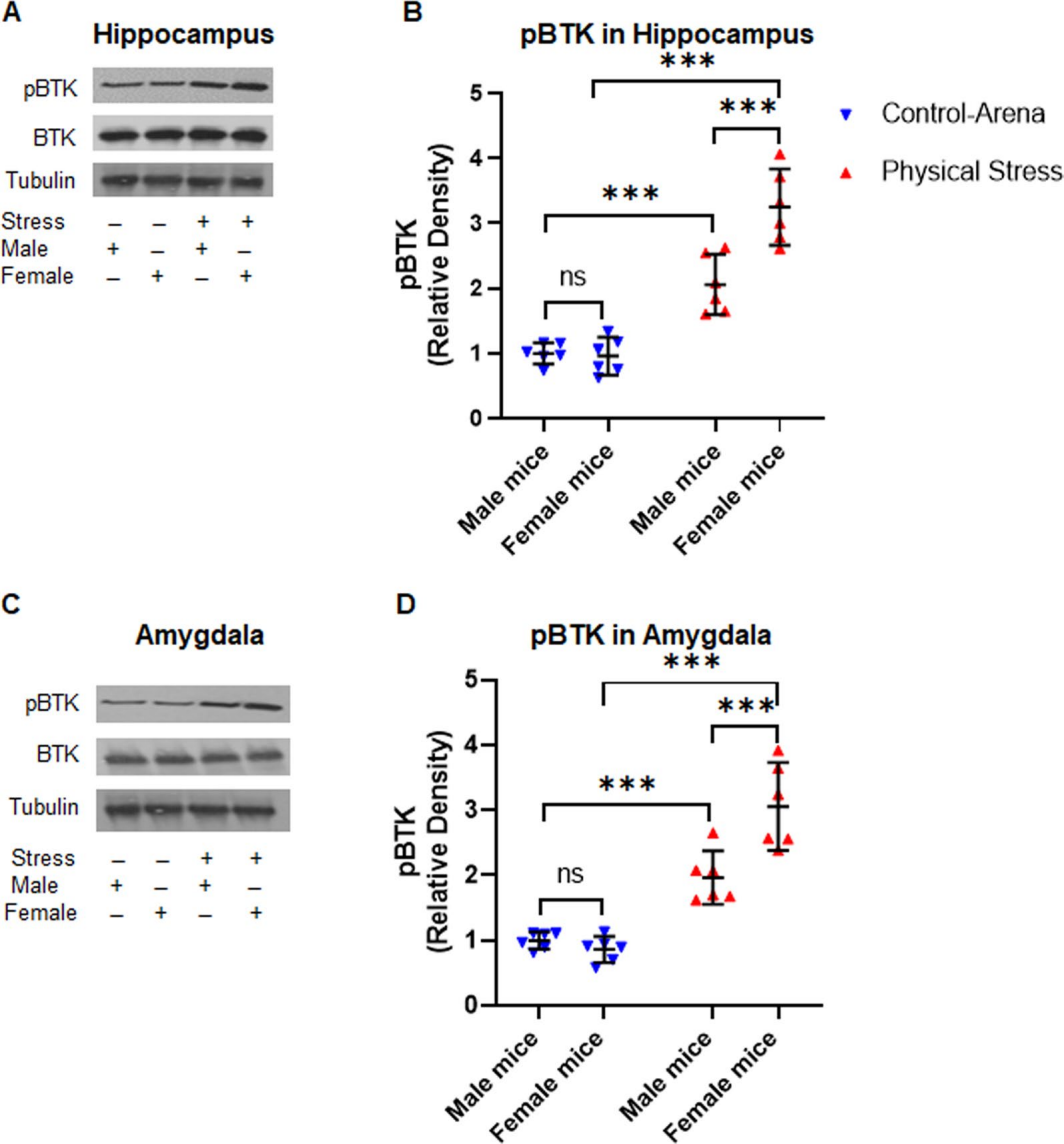
Physical stress in mice induces the activation of BTK in the hippocampus and amygdala. Mice were subjected to physical stress by restraint and underwater trauma. **A** Immunoblot analysis of the hippocampal homogenates using anti-phospho-BTK (pBTK, Tyr223), BTK, and tubulin antibodies. **B** Relative densitometry analysis of pBTK immunoblots showing physical stress significantly induced the activation of BTK, as revealed by increased pBTK levels in stressed mice as compared to controls. **C** Western blot analysis of pBTK, total BTK, and tubulin as a loading control. **D **The relative densitometry analysis of pBTK immunoblots of amygdala samples. The analysis from both hippocampal and amygdala samples showed stressed female mice exhibited significantly increased induction of pBTK relative to their male counterparts and control mice. Data are presented as mean with 95% CI (*n* = 6/group); ***p < 0.001, ns (not significant); two-way ANOVA, followed by Bonferroni post hoc test

To investigate a potential therapeutic avenue, we probed further upstream of NLRP3, and focused on BTK activation, a molecular event that is critical to the assembly and functioning of NLRP3 inflammasome. Phosphorylation of BTK at Tyr223 within the SH3 domain is necessary for the full activation of BTK and its downstream signaling, including induction of NLRP3 inflammasome. We subjected lysates from the hippocampus and amygdala of stressed and control mice to immunoblot analysis, using anti-phospho-BTK antibody (Tyr223, D9T6H, rabbit monoclonal antibody, 1:1000, #87141, Cell Signaling Technology), anti-BTK antibody (D3H5, rabbit monoclonal antibody, 1:1000, #8547, Cell Signaling Technology) and anti-alpha tubulin for loading control (ab4074, rabbit polyclonal antibody, 1:5000, Abcam). On conducting a 2X2 ANOVA (stress, sex) of tubulin-normalized pBTK relative densitometry from the hippocampal lysates revealed a significant main effect of stress[*F* (*1,20*) = 110.63, *p* < 0.001, *η*_*p*_^2^ = 0.85]and a significant main effect of sex [*F* (*1,20*) = 13.03, *p* = 0.002, *η*_*p*_^2^ = 0.4]. This was further qualified by a significant interaction between the two factors [*F* (*1,20*) = 14.97, *p* < 0.001, *η*_*p*_^2^ = 0.43; Fig. 5A, B]. Bonferroni-corrected post hoc testing revealed that physical stress induced an approximately 2- and 3-fold increase in phospho-BTK in male and female mice, respectively (M: 2.058 ± 0.44, F: 3.25 ± 0.56; *t* = 5.29, *p* < 0.001), compared to control male and female mice (M: 1.00 ± 0.16, F: 0.96 ± 0.28; *t* = 998, *p* = 1.00). Between the two experimental conditions the upregulation in phospho-BTK was much starker in stressed females as compared to control female mice (*t* = 10.17, *p* < 0.001) than stressed males and control male mice (*t* = 4.70, *p* = 0.002), although both sexes demonstrated a significant upshot of phospho-BTK following stress. There was no change in total BTK levels in all the groups, as revealed by immunoblots probed with BTK antibody. Very similar patterns and effect sizes were found in the amygdala samples (Fig. 5C, D). Immunoblot analysis of amygdala samples revealed that phospho-BTK was upregulated following physical stress, but disproportionately more significantly in females than male mice. Thus far, these experiments provided the evidence that BTK, an upstream regular of NLRP3, is activated in hippocampus and amygdala of mice following physical stress in sexually divergent pattern.

### Pharmacological inhibition of BTK produces anxiolysis and attenuates NLRP3, Caspase 1 and IL1β upregulation in hippocampus and amygdala of stressed mice

Several recent studies in different disease models, such as ischemic brain injury, cardiac dysfunction, and inflammatory diseases have suggested that BTK is an upstream positive-regulator of NLRP3 inflammasome, which in turn induce downstream proinflammatory molecular events such as activation of Caspase 1 and IL1β [36, 59–62]. Utilizing ibrutinib, as an FDA approved BTK inhibitor, these studies have suggested BTK as a therapeutically relevant NLRP3 regulator. Ibrutinib binds irreversibly to BTK and inhibits the phosphorylation of Tyr 223, thus blocking BTK activity. In a proof-of-concept study, we asked if inhibiting BTK with ibrutinib would provide protection in physical stress model. We injected ibrutinib (3 mg/kg, i.p.) into control groups of mice and as well as those subjected to restraint and underwater trauma, and assessed them on behavioral and molecular end points.

**Fig. 6:**
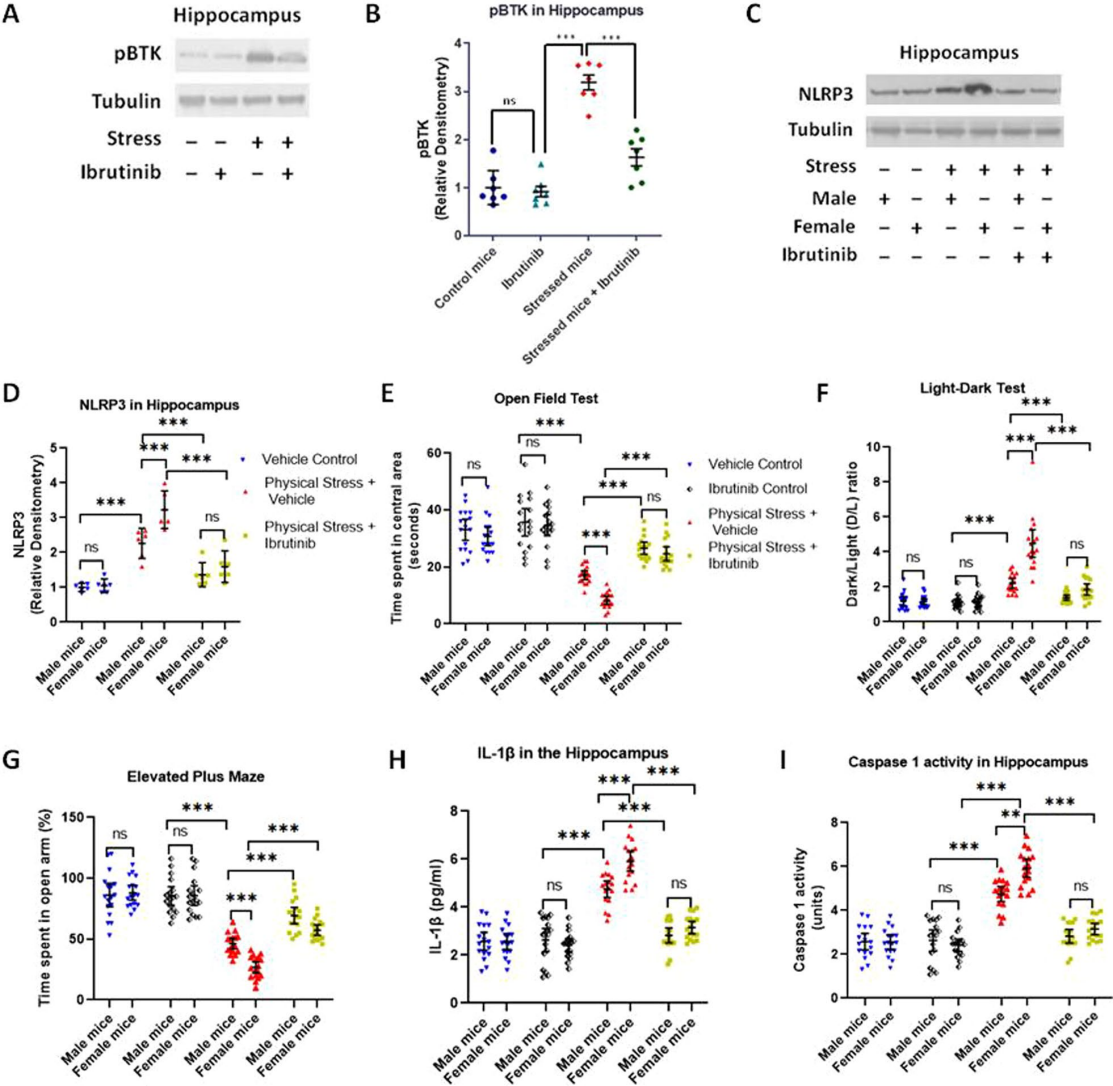
Pharmacological inhibition of BTK with ibrutinib attenuates hyper-anxious behavior as well as proinflammatory molecules in the hippocampus of stressed mice. Mice subjected to restraint stress and underwater trauma was injected with ibrutinib (3 mg/kg, i.p.) or vehicle. **A** Immunoblot analysis of pBTK in hippocampal homogenates. **B** The relative densitometry of pBTK immunoblots from hippocampal samples showing ibrutinib treatment significantly reduced the pBTK levels in physically stressed mice as compared to vehicle-treated stressed mice. All values are presented as mean with 95% CI (*n* = 6/group); ***p < 0.001, **p < 0.01 ns (not significant); 2X2 factorial ANOVA followed by Bonferroni post hoc test. **C** Immunoblot analysis of NLRP3 in hippocampus homogenates **D **The relative densitometry of NLRP3 immunoblots from hippocampal samples showing ibrutinib treatment significantly reduced the NLRP3 inflammasome levels in physically stressed mice as compared to vehicle-treated stressed mice. ***p < 0.001, ns (not significant); One way ANOVA, followed by Bonferroni post hoc test **E** Open field test: physically stressed mice dosed with ibrutinib exhibited significantly decreased anxiety levels when compared to the vehicle controls, as illustrated by increased exploration time in the central area of the OFT**. F** Light–dark test: physically stressed mice dosed with ibrutinib exhibited significant rescue from hyper-anxiety as explained by the significant increase in their time spent in the light chamber of the LDT (**G**). Elevated plus maze test: physically stress mice injected with ibrutinib displayed significantly reduced anxiety, as evidenced by the increased duration of time spent in the open arms of the EPM. **H** Analysis of IL1β in the hippocampus by ELISA revealed physically stressed mice dosed with ibrutinib showed diminished proinflammatory IL1β. **I** Caspase 1 activity in the hippocampus: administration of ibrutinib in physically stressed mice showed reduced Caspase 1 activation, elucidating significant rescue from the anxiogenic proinflammatory pathway. All values are presented as mean with 95% CI (*n* = 22/group); ***p < 0.001, ns (not significant); 2X2X2 factorial ANOVA, followed by Bonferroni post hoc test

First, we tested if ibrutinib indeed lowers BTK activation, in the hippocampal lysates of female animals which by far show greater pathology and anxious behavior, by running anti-phospho-BTK immunoblots and comparing the relative densities of phospho-BTK1 normalized to tubulin (Fig. 6A, B). A 2X2 ANOVA with stress and drug as factor showed main effects of both [ stress: *F* (*1,24*) = 95.68, *p* < 0.001, *η*_*p*_^2^ = 0.8; sex: F (*1,24*) = 30.67, *p* < 0.001, *η*_*p*_^2^ = 0.56] and a significant interaction [F (*1,24*) = 24.82, *p* < 0.001, *η*_*p*_^2^ = 0.51]. Ibrutinib treatment lowered the levels of phospho-BTK in the hippocampi (mean difference: 1.56) of stressed female mice compared to vehicle-treated stressed females (*t* = 7.44, *p* < 0.001; Fig. 6B). Second, we wanted to see if ibrutinib-injected stressed animals demonstrated a change in the levels of NLRP3 inflammasome, a key mediator for downstream inflammatory cascade. To this end we isolated hippocampi from stressed animals treated with vehicle or ibrutinib and subjected the hippocampal lysates to Western blot analysis (Fig. 6C). We observed that ibrutinib inhibited the induction of NLRP3 in physically stressed mice, as compared to control group of mice. We compared the relative density of NLRP3 normalized with tubulin and analyzed the groups by a one-way ANOVA test (Fig. 6D). The group means were significantly different [*F* (5,30) = 33.95, *p* < 0.001, *η*^2^ = 0.85]. Bonferroni-corrected post hoc testing revealed significantly high upregulation of NLRP3 in stressed females as compared to their male counterparts (*p* < 0.001). Further, the relative density of NLRP3 in ibrutinib-treated stressed male and female mice was significantly reduced as compared to their stressed counterparts administered with only vehicle control (*p* < 0.001).

Next, we carried out behavioral analysis on control and stressed animals and analyzed them by sex as well as drug treatment. For each experiment, we used separate 2(stressed, control) X 2 (male, female) X 2(vehicle, ibrutinib) Factorial ANOVAs. We used same three behavioral tests described above. When the time spent in the central square of open field test was analyzed by a factorial ANOVA (Fig. 6E), there was a significant main effect of stress [*F* (*1,128*) = 193. 19, *p* < 0.001, *η*_*p*_^2^ = 0.6], a main effect of sex [*F* (*1,128*) = 11.63, *p* < 0.001, *η*_*p*_^2^ = 0.08], and a significant main effect of drug [*F* (*1,128*) = 60.654, *p* < 0.001, *η*_*p*_^2^ = 0.32]. The interaction between stress and drug was significant with the largest effect size [*F* (*1,128*) = 21.202, *p* < 0.001, *η*_*p*_^2^ = 0.14]. Control mice both vehicle treated (M: 33.06 s ± 7.24, F: 30.71 s ± 6.45; *t* = 1.16, *p* = 1.00) or ibrutinib treated (M: 35.71 s ± 9.38, F: 34.65 s ± 7.19; *t* = 0.51, *p* = 1.00) did not exhibit significant difference within sex or treatment (M: *t* = 1.27, p = 1.00; F: *t* = 1.92, *p* = 1.00). Vehicle-treated mice subjected to physical stress, however, displayed a sharp decline in the time spent in the central square of the open field, more prominently in the females (M: 17.12 ± 3.04, F: 8.35 ± 2.83; *t* = 4.235, *p* < 0.001) compared to control animals, both vehicle treated (M: *t* = 7.703, *p* < 0.001; F: *t* = 10.8, *p* < 0.001) as well as ibrutinib treated (M: *t* = 8.98, p < 0.001; F: *t* = 12.71, *p* < 0.001). Ibrutinib-treated mice subjected to physical stress showed significant rescue from hyper-anxious behavior as compared to vehicle-treated stressed mice in both males (M: 26.53 s ± 4.16, *t* = 4.548, *p* < 0.001.) and females (F: 24.59 s ± 4.77; *t* = 7.85, *p* < 0.001). After ibrutinib treatment, physically stressed males and females spent similar time in the central square of the open field (*t* = 0.94, *p* = 1.00).

The light–dark test showed a similar pattern (Fig. 6F) in ibrutinib-treated mice. The factorial ANOVA conducted on the D/L ratios revealed a significant main effect of stress [F (*1,128*) = 60.23, *p* < 0.001, *η*_*p*_^2^ = 0.50], a main effect of sex [*F* (*1,128*) = 33.98, *p* < 0.001, *η*_*p*_^2^ = 0.21], and a significant main effect of drug [*F* (*1,128*) = 56.62, *p* < 0.001, *η*_*p*_^2^ = 0.31]. This was further qualified by significant interaction terms, including a 3-way interaction between stress, sex and drug [*F* (*1,128*) = 15.81, *p* < 0.001, *η*_*p*_^2^ = 0.11]. Control mice both vehicle treated (M: 1.19 ± 0.48, F: 1.16 ± 0.33) or ibrutinib treated (M: 1.12 ± 0.38, F: 1.15 ± 0.26) did not exhibit significant difference within sex or drug treatment. Vehicle-treated mice subjected to physical stress, however, showed a markedly increased D/L ratio, with female ratios being significantly higher (M: 2.21 ± 0.54, F: 4.48 ± 1.53; *t* = 9.68, *p* < 0.001) compared to control unstressed animals, both vehicle treated (M: *t* = 4.36, p < 0.001; F: *t* = 14.17, *p* < 0.001) as well as ibrutinib treated (M: *t* = 3.49, p = 0.02; F: *t* = 14.22, *p* < 0.001). The D/L ratio was significantly reduced in physically stressed mice treated with ibrutinib, as compared to vehicle-treated stressed mice (M: 1.5 ± 0.29, F: 1.86 ± 0.62, *t* = 1.9, *p* = 1.00). As compared to controls, ibrutinib clearly improved the anxious behavior in stressed mice as evident by increased time spent in light chamber of the light–dark test (*p* < 0.001).

Next, we analyzed the data from elevated plus maze test (Fig. 6G). The factorial ANOVA conducted on the EPM data revealed a significant main effect of stress [*F* (*1,128*) = 268.113, *p* < 0.001, *η*_*p*_^2^ = 0.68], a significant main effect of sex [*F* (*1,128*) = 11.06, *p* = 0.001, *η*_*p*_^2^ = 0.08] and a significant main effect of drug [*F* (*1,128*) = 32.27, *p* < 0.001, *η*_*p*_^2^ = 0.2]. This was further qualified by significant interaction terms, with the most significant interaction between stress and drug [*F* (*1,128*) = 40.33, *p* < 0.001, *η*_*p*_^2^ = 0.24]. Control mice, both vehicle treated (M: 28.69 ± 6.21, F: 29.32 ± 4) or ibrutinib treated (M: 28 ± 3.1, F: 28.36 ± 6.29) spent comparable times in the open arms and did not exhibit significant difference within sex or drug treatment (*t* = 0.99, *p* = 1.00). Vehicle-treated mice subjected to physical stress, however, showed a markedly reduced time spent in the open arm, with females spending significantly lesser time in the open arm of the EPM (M: 15.44 ± 2.82, F: 8.9 ± 3.04) compared to control animals, both vehicle treated (*t* = 16.07, *p* < 0.001) as well as ibrutinib treated (M: *t* = 15.59, *p* < 0.001). Treatment with ibrutinib significantly reduced the anxiety in stressed mice, as evident by significant increase in exploration time in the open arm of EPM, as compared to controls (*p* < 0.001). The percentage time spent in the open arm of EPM was markedly improved in both male and female stressed mice treated with ibrutinib (M: 23.08 ± 4.33, F: 19.08 ± 2.81).

Thus far, our model of physical stress produced robust anxious behavior in mice, which was significantly more pronounced in female mice. When we inhibited BTK, an upstream regulator of NLRP3 inflammasome, with a pharmacological inhibitor, in all three behavior tests mice showed significant rescue from heightened anxious behavior, validating the implication of BTK pathway in anxiogenesis following physical stress.

We then turned to investigating biochemical changes in the brain following ibrutinib administration. We measured hippocampal IL1β levels with ELISA and analyzed the data with a factorial ANOVA (Fig. 6H). The factorial ANOVA conducted revealed a significant main effect of stress [*F* (*1,128*) = 153.65, *p* < 0.001, *η*_*p*_^2^ = 0.59], a main effect of sex [*F* (*1,128*) = 7.06, *p* = 0.009, *η*_*p*_^2^ = 0.05], and a significant main effect of drug [*F* (*1,128*) = 101.88, *p* < 0.001, *η*_*p*_^2^ = 0.44]. This was further qualified by significant interaction terms, with the most significant interaction between stress and drug [*F* (*1,128*) = 94.64, *p* < 0.001, *η*_*p*_^2^ = 0.43]. Control mice both vehicle treated (M: 2.58 pg/ml ± 0.73, F: 2.55 pg/ml ± 0.62) or ibrutinib treated (M: 2.63 pg/ml ± 0.95, F: 2.41 pg/ml ± 0.55) had similar levels of IL1β in the hippocampus, did not show a sex difference or an upregulation in IL1β production just by the drug treatment (*t* = 0.99, *p* = 1.00). Vehicle-treated mice subjected to physical stress, had significantly elevated hippocampal IL1β levels, with more prominent increase in females (M: 4.75 pg/ml ± 0.67, F: 3.15 pg/ml ± 0.49) compared to control animals, both vehicle treated (*t* = 16.51, *p* < 0.001) as well as ibrutinib treated (*t* = 14.02, p < 0.001). When dosed with ibrutinib, IL1β levels went down significantly in stressed mice, and abolished the difference between males and females, although females tended to still have slightly elevated IL1β in treated animals (M: 2.82 pg/ml ± 0.59, F: 3.15 pg/ml ± 0.5).

We also analyzed the activity of Caspase 1 in the hippocampus, to validate our results in this pharmacological inhibition experiment, and analyzed the data with a Factorial ANOVA. Overall, the pattern of Caspase 1 activity data (Fig. 6I) mirrored the IL1β data closely. Caspase 1 activity rose sharply and significantly in stressed mice which were vehicle treated, however, ibrutinib treatment significantly attenuated the Caspase 1 activity in hippocampus of both male and female stressed mice (*p* < 0.001). Therefore, this experiment provided evidence that the BTK inhibition, thus likely reduction of NLRP3 inflammasome, is crucial part of the neuroinflammatory response seen in this model of physical stress and anxiogenic behavior, with females displaying a more severe phenotype compared to males.

To further validate our observations with ibrutinib treatment, we repeated this experiment with another compound, LFM-A13, also known in the literature for its ability to specifically suppress BTK [63].

To cross-validate the observations from ibrutinib study, control or stressed mice were injected with another BTK-specific inhibitor LFM-A13 (50 mg/Kg, i.p.) or vehicle and subjected to full battery of anxiety behavior tests and biochemical analysis. Our findings from LFM-A13 confirmed the findings from the ibrutinib study (Additional file 1: Fig. S4A–E). Inhibition of BTK with LFM-A13 significantly suppressed the anxiety behavior in both male and female stressed mice, as compared to control groups (*p* < 0.001). Further, LFM-A13 significantly reduced the activation of proinflammatory mediators Caspase 1 and IL1β in hippocampus (*p* < 0.001). In total, our study suggested an important role of BTK in anxiogenic pathway.

**Fig. 7:**
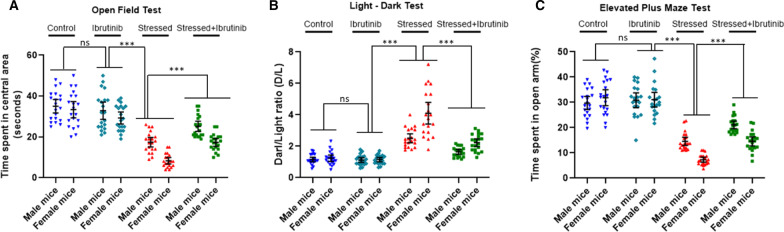
Pharmacological inhibition of BTK with ibrutinib after the induction of stress in mice attenuates hyper-anxious behavior. Mice were injected daily with ibrutinib (3 mg/kg, i.p.) or vehicle two days after restraint stress and underwater trauma. One week after the induction of stress mice were assessed for the hyper-anxious behavior. **A** Open field test: physically stressed mice dosed with ibrutinib after the induction of stress exhibited significantly decreased anxiety levels when compared to the vehicle controls, as illustrated by increased exploration time in the central area of the OFT. **B** Light–dark test: physically stressed mice dosed with ibrutinib after the induction of stress displayed significant rescue from hyper-anxiety as explained by the significant increase in their time spent in the light chamber of the LDT. **C** Elevated plus maze test: physically stress mice injected with ibrutinib after the induction of stress exhibited significantly reduced anxiety, as evidenced by the increased duration of time spent in the open arms of the EPM. All values are presented as mean with 95% CI (*n* = 20/group); ***p < 0.001, ns (not significant); 2X2X2 factorial ANOVA, followed by Bonferroni post hoc test

So far, the experiments gave us proof-of-concept data that inhibition of BTK can lead to a functional rescue of stress-induced anxiogenesis. Next, we tested if treatment with Ibrutinib *post-stress* can cause functional recovery. Ibrutinib treatment two days after stress led to improvement in anxious behavior at 7 days as shown in Fig. 7A–C, using all 3 behavioral tests. We hypothesized once again that the experimental status (stress vs. control), sex (male, female) and drug treatment (vehicle vs. ibrutinib) will determine the behavioral outcome in a pattern similar to as seen with ibrutinib treatment, and there will be significant interaction between one or more factors when analyzed by a 2X2X2 factorial ANOVA.

The factorial ANOVA for the percent time spent in the central quadrant of the open field test revealed significant main effects for all three factors [stress: F (*1,152*) = 238.94, *p* < 0.001, *η*_*p*_^2^ = 0.61; sex: *F* (*1,152*) = 30.713, *p* < 0.001, η_p_^2^ = 0.17; Drug: *F* (*1,152*) = 6.68, *p* = 0.01, *η*_*p*_^2^ = 0.04] and significant interactions between stress and sex [*F* (*1,152*) = 8.46, *p* = 0.004, *η*_*p*_^2^ = 0.05] as well as stress and drug *F* (*1,152*) = 32.672, *p* < 0.001, *η*_*p*_^2^ = 0.18; Fig. 7A]. Overall, the trend was very similar to that observed in Fig. 6C–E. Among vehicle-treated animals, stressed animals spent considerably less time in the central square, [mean difference (MD): 21.3; *t* = 14.97, *p* < 0.001] compared to vehicle-treated control animals. But in ibrutinib-treated animals this difference was considerably less (MD: 8.35; *t* = 5.87, *p* < 0.001), although the treatment did not completely obliterate the difference, which is largely driven by female animals, in whom the effects of stress were much worse.

The factorial ANOVA for the ratio of time spent between the dark and light halves of the apparatus also demonstrated a similar pattern [Fig. 7B]. There were significant main effects for all three factors [stress: *F* (*1,152*) = 198.41, *p* < 0.001, *η*_*p*_^2^ = 0.57; sex: *F* (*1,152*) = 30.82, *p* < 0.001, *η*_*p*_^2^ = 0.17; drug: *F* (*1,152*) = 48.1, *p* < 0.001, *η*_*p*_^2^ = 0.24]. The multi way interaction term between stress, sex and drug treatment was also significant [*F* (*1,152*) = 5.284, *p* = 0.02, *η*_*p*_^2^ = 0.18]. Again, stressed animals spent more time in the dark quadrant compared to vehicle-treated animals, with the effect more pronounced in female animals (MD: 2.86; *t* = 13.86, *p* < 0.001) than males (MD: 1.35; *t* = 6.55, *p* < 0.001). But in ibrutinib-treated animals this difference was not significant in males (MD: 0.52; *t* = 2.52, *p* = 0.19) and considerably less in females, though not obliterated (MD: 1.08; *t* = 5.24, *p* < 0.001).

Finally, we scored the time spent by vehicle vs ibrutinib-treated male and female animals for both stressed and control conditions in the open vs closed arms of the Elevated Plus Maze [Fig. 7C]. The data showed the same pattern. There were significant main effects for all three factors [stress: *F* (*1,152*) = 444.24, *p* < 0.001, *η*_*p*_^2^ = 0.75; sex: *F* (*1,152*) = 13.53, p < 0.001, η_p_^2^ = 0.08; drug: *F* (*1,152*) = 20.34, *p* < 0.001, *η*_*p*_^2^ = 0.03]. There were significant interactions between stress and sex [F (*1,152*) = 26.85, *p* < 0.001, *η*_*p*_^2^ = 0.15] as well as stress and drug *F* (*1,152*) = 18.55, *p* < 0.001, *η*_*p*_^2^ = 0.11]. Again, stressed animals spent less time in the open arms compared to controls, with the effect more pronounced in female animals (MD: 20.53; *t* = 18.57, *p* < 0.001) than males (MD: 12.43; *t* = 11.24, *p* < 0.001). Among stressed animals, ibrutinib-treated animals spent significantly more time in the open arm compared to vehicle-treated animals (MD: 6.89; *t* = 6.234, *p* < 0.001), demonstrating a functional rescue effect. This leads further credence to the idea that ibrutinib can be investigated as a potential therapeutic tool in treatment of stress-induced anxiety.

### The peripheral inflammatory response in the current model of physical stress

**Fig. 8:**
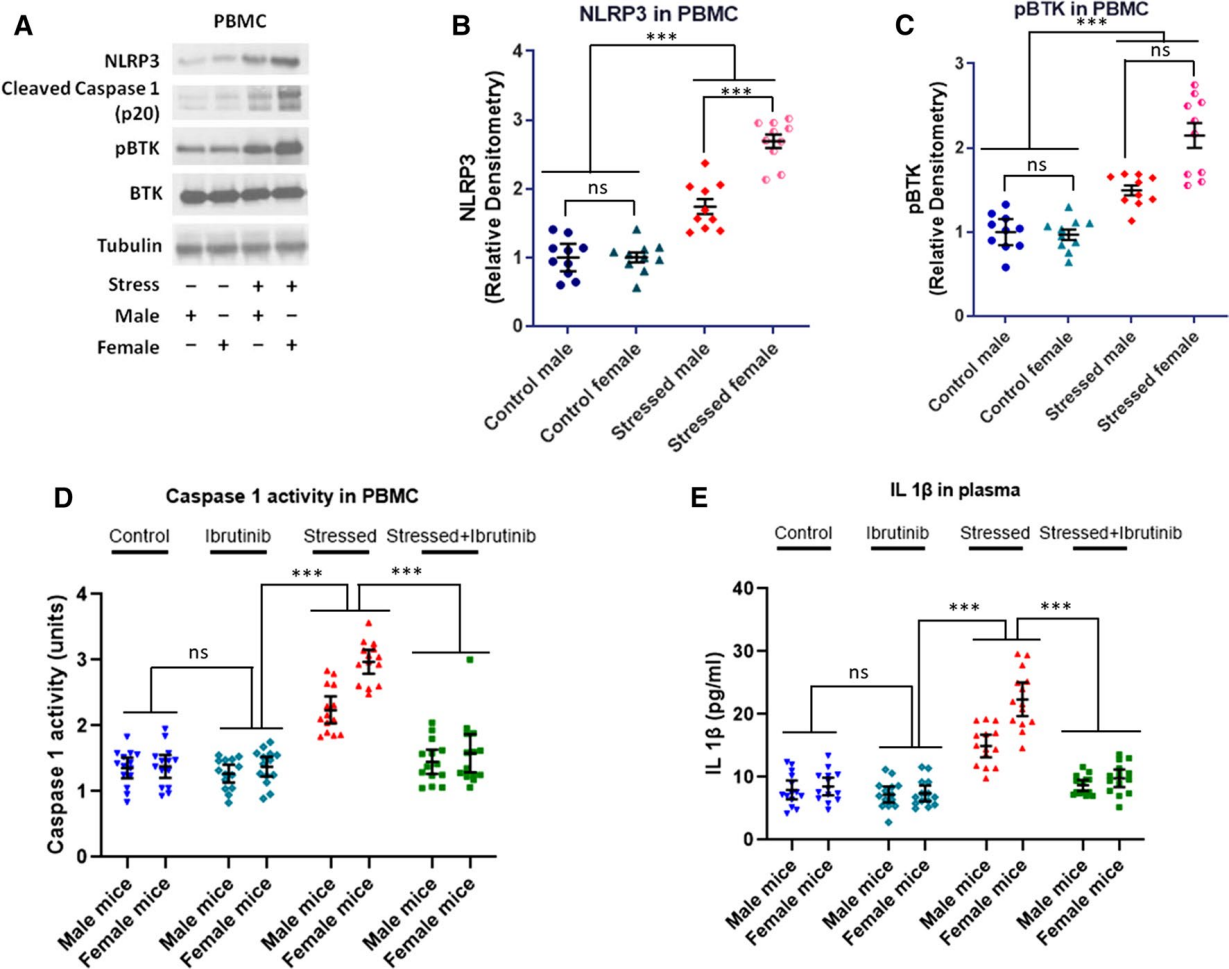
Physical stress in mice induces the activation of pBTK–NLRP3–Caspase 1–IL1β pathway in the peripheral blood mononuclear cell (PBMC). Mice were subjected to physical stress by restraint and underwater trauma. **A** Immunoblot analysis of the PBMC homogenates using anti-NLRP3, cleaved Caspase 1, phospho-BTK (pBTK, Tyr223), BTK, and tubulin antibodies. **B** The relative densitometry of NLRP3 immunoblots of samples from the PBMC homogenates, which shows physical stress-induced higher levels of NLRP3 inflammasome in the PBMC of female mice in comparison to male mice. **C** Relative densitometry analysis of pBTK immunoblots showing physical stress significantly induced the activation of BTK, as revealed by increased pBTK levels in PBMC of stressed mice as compared to controls. **D** Caspase 1 activity in the PBMC homogenates using Caspase 1 assay kit: administration of ibrutinib in physically stressed mice showed reduced Caspase 1 activation, elucidating significant rescue of PBMC from the anxiogenic proinflammatory pathway. **E** Interleukin 1β (IL1β) levels in the plasma revealed physically stressed mice dosed with ibrutinib showed diminished plasma proinflammatory IL1β. All values are presented as mean with 95% CI (*n* = 10–14/group); ***p < 0.001, ns (not significant); 2X2X2 factorial ANOVA followed by Bonferroni post hoc test

It is well documented that the peripheral response to trauma and stress consists of a robust upregulation in inflammatory mediators in clinical and animal studies [68, 69]. Our immunoblot analysis of peripheral blood mononuclear cells (PBMCs) from control and stressed animals demonstrated that the markers we have examined in the brain such as IL-1β, cleaved Caspase 1, NLRP3 and phospho-BTK are all upregulated in the PMBCs post-physical stress (Fig. 8A). The upregulation also follows a sexually divergent response, with inductions of all the above-mentioned being stronger in females compared to males (Fig. 8B, C). A 2 X 2 factorial ANOVA [experimental status (control vs. physical stress), sex (male, female)] analysis of the relative densitometry values from NLRP3 blots demonstrated a main effect of stress [*F* (*1,36*) = 171.57, *p* < 0.001, *η*_*p*_^2^ = 0.83] and stress [*F* (*1,36*) = 26.42, *p* < 0.001, *η*_*p*_^2^ = 0.42] and a significant interaction between stress and sex [*F* (*1,36*) = 26.16, *p* < 0.001, *η*_*p*_^2^ = 0.42], consistent with the sharper rise in NLRP3 in the plasma of female stressed mice, compared to male stressed mice (MD: 0.95; *t* = 5.65, p < 0.001). Phospho-BTK induction post-stress is uniform in both sexes, with only a significant main effect of Stress [F (*1,36*) = 40.84, *p* < 0.001, *η*_*p*_^2^ = 0.53] but not sex [*F* (*1,36*) = 0.66, *p* = 0.42] or the interaction term [*F* (*1,36*) = 0.15, *p* = 0.705]. Post hoc tests revealed a mean difference of 0.47 between controls and stressed animals (*t* = 6.39, *p* < 0.001; Fig. 8C). Treatment with ibrutinib reduces the level of Caspase 1 activity and IL-1β in PBMCs and the plasma, respectively (Fig. 8D, E). A 2X2X2 factorial ANOVA comparing the effects of experimental status (control, physical stress), sex (male, female) and drug treatment (ibrutinib, vehicle) revealed a main effect of all 3 factors [stress: *F* (*1,104*) = 131.13, *p* < 0.001, *η*_*p*_^2^ = 0.558; sex: *F* (*1,104* = 15.94, *p* < 0.001, *η*_*p*_^2^ = 0.04; drug: *F* (*1,104*) = 82.68, *p* < 0.001, *η*_*p*_^2^ = 0.08] and a significant three-way interaction term [*F* (*1,104*) = 7.59, *p* < 0.001, *η*_*p*_^2^ = 0.07]. Physical stress causes an upregulation of Caspase 1 activity, steeper in females (2.5-fold) vs. males (1.73-fold). Ibrutinib-treated stressed animals demonstrate PBMC activity levels of Caspase 1 comparable to the controls (Fig. 8D). Plasma IL-1β levels mirrored the same pattern (Fig. 8E). Overall, our study for the first time demonstrates BTK-mediated activation of systemic and central NLRP3–IL1β pathway in stress paradigm.

## Discussion

The present study investigated the proinflammatory NLRP3–Caspase 1–IL-1β pathway, which recent research has indicated as a key mediator of heightened inflammatory response in several neuropathological conditions. We have used two different models of stress, first, a direct model of single prolonged immobilization stress, and forced swim followed by brief submersion underwater; second, predator odor which was an operationalized form of psychological stress. Both are established stress models in rodents [64]. For behavioral outcomes, we used a set of well validated tests of anxiety in rodents [65–67], in addition to biochemical analysis of the brains of animals. To control for stress on account of relocation to stress behavior mouse facility, we consistently used control animals that were relocated from the animal facility and housed in the room where stress experiments were conducted, yet to prevent vicarious stress, they had no direct view or exposure to the arena or the mice undergoing stress experiments, precluding any olfactory or visual cues to stress.

Our study indicates that both models of stress used elicits a sharp increase in anxious behavior and neuroinflammatory mediators and that this increase in both behavior and inflammatory profile is significantly more pronounced in females compared to males. The presence of a pronounced neuroinflammatory response post-stress is well established in several pre-clinical rodent models and has been reviewed extensively [68, 69]. Elevation of a range of peripheral inflammatory mediators have been consistently observed in PTSD patients compared to age matched controls, both at protein and genetic levels[41, 70–73]. The sexually dimorphic patterns of anxious behavior and the upregulation of neuroinflammatory markers we observed in our model are also consistent with three lines of evidence. First, in humans stress-related disorders affect more women than men [1, 4], as do anxiety related disorders [74]. Second, Lasselin and colleagues in their review of several human experimental stress models has suggested that the neuroinflammatory response in discussed models is overall, more robust in females than males [75]. Finally, the sexually dimorphic neuroinflammatory response with females showing more robust neuroinflammation post-stress is noted in several rodent models of stress [76–79].

The NLRP3 inflammasome has been recently the target of intense research as a molecule of therapeutic interest in Alzheimer’s disease [80, 81], amyotrophic lateral sclerosis [82], vascular dementia [30], multiple sclerosis and experimental autoimmune encephalopathy[83–85], stroke [26, 86], bacterial meningitis [22, 87], traumatic brain injury [88], cerebral hemorrhage [89], in addition to systemic illnesses such as peritoneal fibrosis [90] and type-2 diabetes [91], in both clinical and pre-clinical studies. The NLRP3 inflammasome has also received attention in the literature pertaining to psychiatric disorders [28, 29]. The activation of the NLRP3 inflammasome has been observed in the mononuclear blood cells from patients with major depressive disorder [92]. Due to an established literature on the elevation of inflammatory markers following stress, as well as the documented role of the NLRP3 inflammasome activation in the maturation of IL1β, a powerful cytokine that has often been regarded as a “master regulator” of the neuroinflammatory response, the NLRP3 activation has also been proposed as a valuable candidate for therapeutic exploitation in stress-related disorders [93]. Preclinical models of stress-induced depression, or foot-shock based stress models have utilized interfering with NLRP3 inflammasome activation using genetic knockout[33, 94, 95] as well as pharmacological inhibition using several different pharmacological inhibitors [96, 97]. The suppression of NLRP3 activation has led to alleviation of behavioral deficits in the said models. In that, our results are consistent with the existing literature. However, while we have validated the trends in literature in our work, there are several novel aspects of our work. We inhibited NLRP3 with MCC950, one of the most potent and specific small molecule inhibitors in the context of a single prolonged stress episode as well as psychological stress. We have performed these experiments in both female and male mice, which are distinct from considerable bulk of research in this field, done exclusively on male animals. For the first time, we have demonstrated the sexually divergent induction of NLRP3. We demonstrated the heightened activation of proinflammatory NLRP3–Caspase 1–IL1β pathway in the hippocampus and amygdala of stressed mice, which was significantly more pronounced in females as compared to their male counterparts. We also demonstrated an attenuated neuroinflammatory response in terms of IL1β, Caspase 1 activity concomitant to MCC950 based NLRP3 inhibition consistent with anxiolysis in both female and male animals.

We further probed upstream of the NLRP3 inflammasome and zeroed in on Bruton’s tyrosine kinase that acts as a positive regulator of NLRP3 activation [35, 36, 60]. These studies demonstrated that BTK is required for NLRP3-mediated maturation of IL1β from pro-IL1β in macrophages. For the first time, our study provided evidence that stress induces the activation of BTK in both hippocampus and amygdala, further in a sexually dimorphic way, with female showing much more pronounced pBTK levels in brain. Several earlier studies made use of ibrutinib, which is known to rapidly penetrate the BBB [105, 106], selectively inhibits BTK and is already FDA approved for oral clinical use in certain types of lymphomas and leukemia [37, 38, 98–100]. It is a highly potent small molecule inhibitor that selectively binds to cysteine 481 residue in the allosteric inhibitory segment of BTK kinase domain, thus irreversibly inhibits its full activation by blocking its autophosphorylation at tyrosine residue 223 [107].

Using a murine model of modified middle cerebral artery occlusion for 60 min followed by reperfusion, Ito and colleagues demonstrated that ibrutinib treatment for up to 12 h post-reperfusion minimized infarct volume, improved neurological scores at recovery and markedly attenuated levels of the proinflammatory cytokines IL1β, IL6 and TNF-α [101]. In our study, we observed that ibrutinib-treated animals displayed an almost 40–50% rescue in anxious behaviors and a concomitant reduction in Caspase 1 activity and IL1β, in both hippocampus and amygdala, which was more pronounced in female animals. Since, we wanted to further validate our findings with BTK inhibition, we repeated the pharmacological inhibition experiment with LFM-A13, a second selective BTK inhibitor [102–104] in the single prolonged stress model. The results closely mirrored that from the ibrutinib experiment, further suggesting a potential role of BTK in modulating anxious behavior and proinflammatory pathway. To make the proof-of-concept data more relevant to application in a clinical setting, we inhibited BTK with ibrutinib post-stress. We found that systemic injection of ibrutinib at 2 days after the induction of stress in mice resulted in attenuation of the anxious behavior, as measured by open field test, light–dark tests and elevated plus maze test. This indicates that ibrutinib could be investigated further as a promising candidate in the treatment of stress disorders. Ibrutinib (Imbruvica) has been approved for the treatment of certain types of cancers, however, there are several side effects, such as diarrhea, arterial fibrillation and bleeding, which have been partially attributed to off-target effects on the epidermal growth factor receptor and the Tec family proteins other than BTK [108, 109]. Though we could not discount the possibility of off-target effects of ibrutinib in our study, we also used another small molecular specific BTK inhibitor LFM-A13 and confirmed that BTK inhibition provided protection from hyper-anxious behavior following induction of stress in mice.

Several studies in the past have tried to decipher the cellular correlates of neuroinflammation associated with stress disorders. Jones et al. [110] demonstrated that stress-induced IL1β primarily localized in astrocytes of hippocampus and intra-dorsal hippocampal infusion of IL1β receptor antagonist inhibited stress-induced fear learning. Further, following 48 h after induction of stress hippocampal microglial marker Iba-1 was reduced, whereas astrocyte marker GFAP was unaltered. In contrast to this, Dong et al. recently reported significant increase in Iba-1 microglia numbers in hippocampus following 72 h of electric foot shocks [95]. Further, in microglia isolated from animals 3-h post-foot shock they detected upregulation of IL1β transcripts. Several studies in past have demonstrated the expression of NLRP3 in microglia, astrocytes and neurons [111–113], however, in a study of animal models of depressive disorder expression of NLRP3 was reported only in microglia [114].

A growing body of evidence indicates a significant association between peripheral proinflammatory markers such as plasma IL1β in stress and trauma-related disorders [39, 41, 115, 116]. PBMCs isolated from subjects with PTSD also exhibit significantly higher spontaneous production of IL1β and other proinflammatory cytokines, which correlate well with the severity of the PTSD symptoms [40]. We observed that PBMCs isolated from stressed mice showed significantly higher levels of pBTK, NLRP3 and cleaved caspase 1. In addition, we found significantly enhanced level of IL1β in the plasma of stressed mice. Identification of transcripts with overlapping expression profile between blood and brain in stress disorders suggests the importance of peripheral inflammation in stress disorders [115, 117]. Recent studies have shown that increased peripheral inflammation can predict altered functional connectivity in the major brain regions related to anxiety in the depression and PTSD [116, 118]. Through there is a wide consensus that a few of the inflammatory markers, especially IL1β, are enhanced in blood following stress, however, bi-directional relationship, i.e., if peripheral inflammation contributes to stress disorders is not well understood [11, 119, 120]. Since inflammation is also known to alter the properties of the blood brain barrier in general, it is quite plausible that infiltrating peripheral cells partially drive the central effects. Teasing apart the exact contribution of the peripheral versus central immune response will require more in-depth studies. Nevertheless, our results show that the ibrutinib administration in stressed mice attenuates the IL1β in plasma and Caspase 1 activity in PBMCs, indicating that ibrutinib regulates peripheral immune responses in addition to its central effect.

## Conclusion

In summary, we present novel and robust proof-of-concept findings, and provide the first evidence of BTK as a key driver of anxiogenesis and proinflammatory NLRP3–IL1β pathway in a rodent model of stress. We demonstrated sexually divergent activation of BTK, which was significantly more in stressed females as compared to their male counterparts. Inhibition of BTK with specific inhibitors ibrutinib and LFM-A13 resulted in significant reduction of proinflammatory NLRP3 inflammasome and IL1β, suggesting that BTK inhibition interferes with NLRP3 activation and limits Caspase 1 activity, ultimately lowering the levels of IL1β. This is also to our knowledge, the first in vivo therapeutic application of ibrutinib and LFM-A13 in a stress model, which clearly demonstrated anxiolytic effect of these BTK inhibitors in stressed mice. Stress leads to an exacerbated neuroinflammatory response and significantly more anxiety in female mice, and with both these BTK inhibitors the rescue in female animals were also more pronounced than in males.

Ibrutinib, which is already FDA approved for use in certain types of cancers, can be explored further as a potential therapeutic molecule, as well as further development of LFM-A13 or other BTK inhibitors. Especially for ibrutinib, a chemotherapeutic drug, side effect and tolerance data have been reviewed extensively [121]. However, the drug has been administered in patients of central nervous system lymphoma [99], and recently in patients of severe COVID-19 to prevent the initiation of the cytokine storm [121], suggesting a careful titration of dosage under monitored condition can theoretically be conceived for other conditions. Trauma and stress-related disorders, comorbid with depression and anxiety is a major cause of debilitating psychiatric illness, which is already being indicated as a massive challenge due to the stressful situations globally created at the wake of the COVID-19 pandemic [122, 123]. Urgent research is needed to find feasible therapeutic alternatives beyond the accepted SSRI–SNRI-based treatment regimens for such disorders. In this context, further studies are required to explore the potential of BTK as a novel target for the treatment of PTSD and anxiety disorders.

## Supplementary Information

**Additional file 1: Figure S1.** Female mice exhibit exacerbated anxiety following predator odor stress. **Figure S2.** Physical stress in mice leads to induction of cleaved Caspase 1 in amygdala and hippocampus. **Figure S3. **Inhibition of NLRP3 inflammasome by treating stressed mice with MCC950 attenuates IL1β and Caspase 1 activity in amygdala. **Figure S4. **Inhibition of BTK with LFM-A13 in physically stressed mice provided protection from hyper-anxious behavior and inhibited the proinflammatory pathway.

## Data Availability

Not applicable.

## Abbreviations

ANOVA: Analysis of variance
BTK: Bruton’s tyrosine kinase
DMSO: Dimethyl sulfoxide
ELISA: Enzyme linked immunosorbent assay
EPM: Elevated plus maze test
IL1β: Interleukin-1 beta
IL-6: Interleukin-6
LDS: Lithium dodecyl sulfate
LDT: Light–dark test
LFMA13: 2-Cyano-N-(2,5-dibromophenyl)-3-hydroxy-2-butenamide
MCC950: 1,2,3,5,6,7-Hexahydro-s-indacen-4-ylcarbamoyl-[4-(2-hydroxypropan-2-yl)furan-2-yl]sulfonylazanide
NLRP3: Nucleotide-binding oligomerization domain (NOD) like receptor (NLR) family, pyrin domain containing protein 3
OFT: Open field test
PAGE: Poly-acrylamide gel electrophoresis
PBS: Phosphate-buffered saline
PBMC: Peripheral blood mononuclear cells
PTSD: Posttraumatic stress disorder
SSRI: Selective serotonin reuptake inhibitors
SNRI: Serotonin–norepinephrine reuptake inhibitors
TBST: Tris-buffered saline with Tween
TNF α: Tumor necrosis factor alpha
