# Supplementary material for: Bruton’s tyrosine kinase drives neuroinflammation and anxiogenic behavior in mouse models of stress
Source: J Neuroinflammation. 2021 Dec 11;18:289. doi: 10.1186/s12974-021-02322-9 (PMC8665324; doi:10.1186/s12974-021-02322-9)
Supplement: Supplementary file 1 — Additional file 1: Figure S1. Female mice exhibit exacerbated anxiety following predator odor stress. Figure S2. Physical stress in mice leads to induction of cleaved Caspase 1 in amygdala and hippocampus. Figure S3. Inhibition of NLRP3 inflammasome by treating stressed mice with MCC950 attenuates IL1β and Caspase 1 activity in amygdala. Figure S4. Inhibition of BTK with LFM-A13 in physically stressed mice provided protection from hyper-anxious behavior and inhibited the proinflammatory pathway. [file 12974_2021_2322_MOESM1_ESM.docx]

**Additional file 1**

**Figure S1**


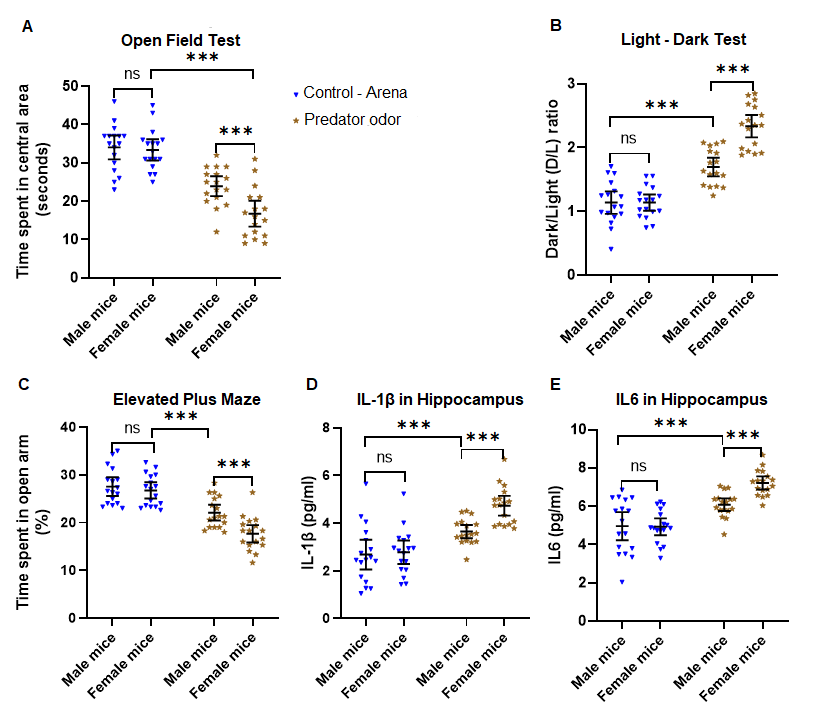
**Figure S1. Female mice exhibit exacerbated anxiety following predator odor stress. (A)** Evaluation of anxious behavior of stressed (predator odor) and non-stressed control mice using open field test (OFT). Female mice exposed to predator odor exhibited significantly higher anxiety levels when compared to stressed males and controls, as depicted by their hesitation to explore the central area of the OFT. **(B)**The light-dark test showed female mice exposed to predator odor exhibited significantly higher anxiety levels (higher Dark-Light ratios) when compared to the male stressed mice and control, as demonstrated by their reluctance to spend more time in the light chamber of the LDT. **(C)** Elevated plus maze test showed female mice subjected to predator odor displayed significantly increased anxiety levels compared to the other groups, as evidenced by their avoidance to spend more time in the open arms of the EPM. **(D)** Evaluation of IL1β levels by ELISA in hippocampal homogenates revealed female mice exposed to predator odor showed aberrantly higher IL1β as compared to stressed males. **(E)** Exposure of predator odor to female mice caused aberrantly higher IL6 levels in the hippocampus as compared to their male counterparts. All data are presented as Mean with 95% CI (n=17/group); ***p<0.001, ns (not significant); one-way *ANOVA* followed by Bonferroni *post-hoc* test.

**Figure S2**


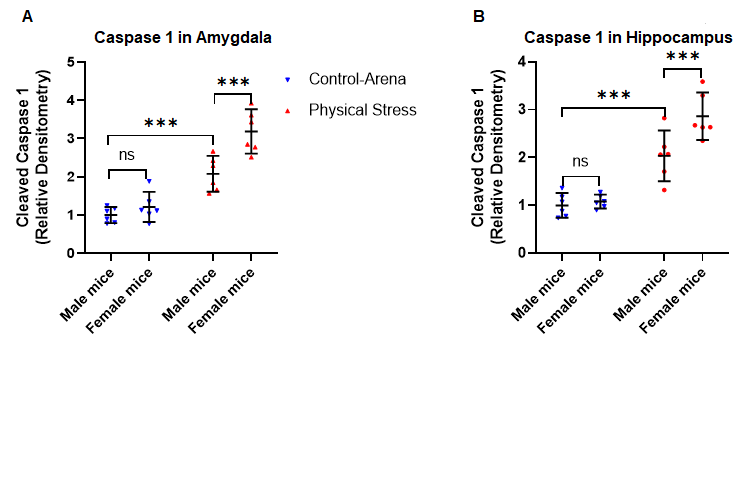
**Figure S2. Physical stress in mice leads to induction of cleaved Caspase 1 in amygdala and hippocampus.** Physical stress was induced by subjecting mice to restraint and underwater trauma. **(A)** Analysis of amygdala homogenates for cleaved Caspase 1 (p20) using quantitative densitometry of immunoblots. **(B)** Relative densitometry analysis of cleaved Caspase 1 immunoblots from hippocampal homogenates. Mice subjected to restraint and underwater trauma show upregulation of Caspase 1 activity, as evident by the increased level of cleaved Caspase 1 in both amygdala and hippocampus of stressed mice. All data are presented as Mean with 95% CI (n=6/group); ***p<0.001, ns (not significant); one-way *ANOVA* followed by Bonferroni *post-hoc* test.

**Figure S3**


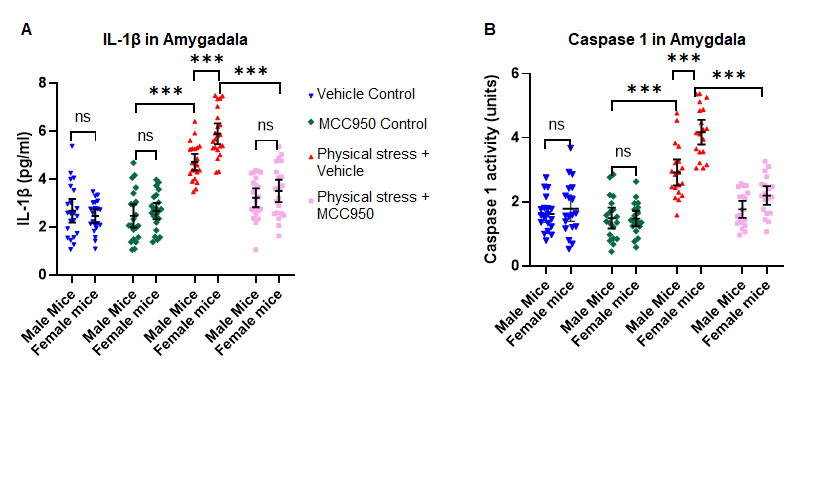
**Figure S3. Inhibition of NLRP3 inflammasome by treating stressed mice with MCC950 attenuates IL1β and Caspase 1 activity in amygdala**. Physical stress was induced by subjecting mice to restraint and underwater trauma. Control mice were treated with either vehicle or MCC950. Similarly, stressed mice were treated with either vehicle or MCC950. **(A)** Analysis of IL1β in amygdala homogenates by ELISA revealed mice exposed to physical stress showed aberrantly higher IL1β compared to control mice. Treatment of stressed mice with MCC950 showed a significant reduction of IL1β. **(B)** Caspase 1 activity in amygdala: intraperitoneal administration of MCC950 in physically stressed mice showed reduced Caspase 1 activation, elucidating key role of NLRP3 in anxiogenic Caspase 1 - IL1β pathway. Caspase 1 activity in amygdala homogenates was measured by Caspase 1 activity assay kit. All values are presented as Mean with 95% CI (n=19-22/group); ***p<0.001, ns (not significant); 2X2X2 factorial ANOVA followed by Bonferroni *post-hoc* test.

**Figure S4**


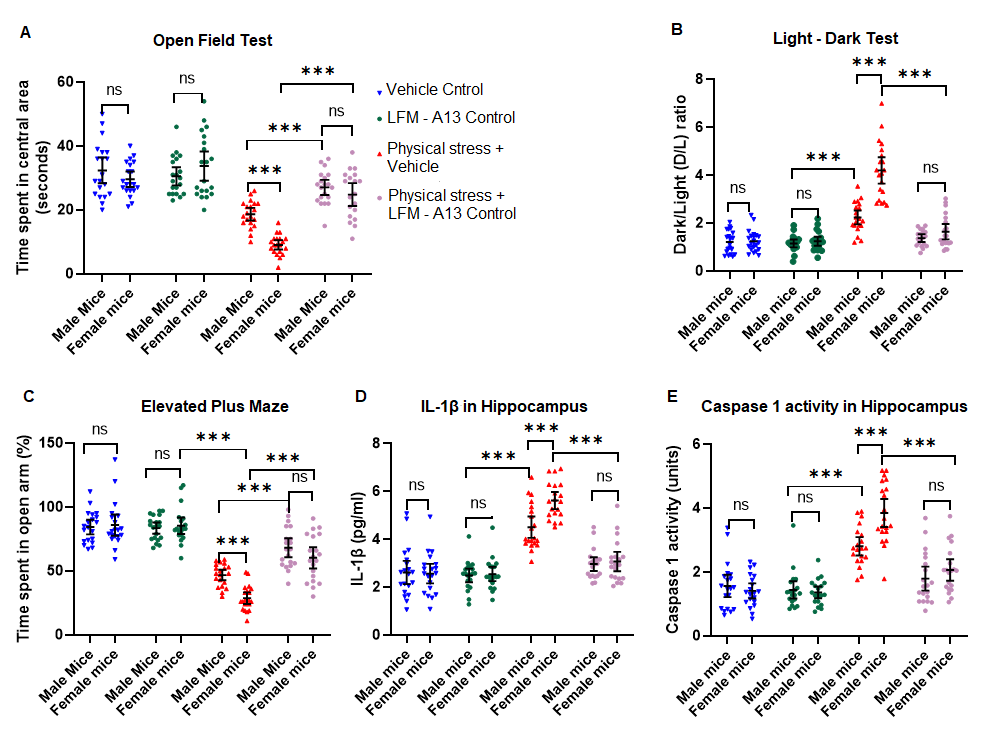


**Figure S4. Inhibition of BTK with LFM-A13 in physically stressed mice provided protection from hyper-anxious behavior and inhibited the proinflammatory pathway. (A)** Analysis of the open field test, following the administration of LMF-A13 in physically stressed mice, there was evidence of significantly decreased anxiety level, as illustrated by the increase in the time of exploration in the central area of the OFT. **(B)** Light-Dark test: physically stressed mice dosed with LMF-A13 showed significant rescue from anxiety, as illustrated by the significant increase in time to spend in the light chamber of the LDT. **(C)** Results of an elevated plus maze also revealed physically stressed mice dosed with LMF-A13 displayed a significant reduction in anxiety, as evidenced by the increase in the duration of time spent in the open arms of the EPM. **(D)** Examination of IL1β levels by ELISA in the hippocampus revealed physically stressed mice dosed with LMF-A13 showed diminished IL1β levels. **(E)**Caspase 1 activity in the hippocampus: physically stressed mice administered with LMF-A13 showed reduced Caspase 1 activation, elucidating significant rescue from anxiogenic proinflammatory pathway. All values are presented as Mean with 95% CI (n=20/group); ***p<0.001, ns (not significant); 2X2X2 factorial ANOVA followed by Bonferroni *post-hoc* test.
